# The effect of bone marrow mesenchymal stem cell-derived extracellular vesicles on bone mineral density and microstructure in osteoporosis: A systematic review and meta-analysis of preclinical studies

**DOI:** 10.1371/journal.pone.0327011

**Published:** 2025-06-30

**Authors:** Ying Zhang, Xining Xu, Ximei Ren, Zhenghong Li

**Affiliations:** 1 Department of Orthopedics, The Affiliated Hospital Southwest Medical University, Sichuan, China; 2 Nursing Department, 940th Hospital of the Joint Logistics Support Force of the PLA, Gansu, China; 3 Department of Rehabilitation Medicine, 940th Hospital of the Joint Logistics Support Force of the PLA, Gansu, China; 4 Department of General Practice, 940th Hospital of the Joint Logistics Support Force of the PLA, Gansu, China; Università degli Studi della Campania, ITALY

## Abstract

**Objective:**

The treatment of osteoporosis is challenged by limited bone regeneration and side effects. Bone marrow mesenchymal stem cell-derived extracellular vesicles (BMSC-EVs) have gained widespread attention as a potential therapeutic approach. This study aims to evaluate the effects of BMSC-EVs on bone density, trabecular microstructure, and biomechanical properties in animal models of osteoporosis, providing evidence to support clinical translation and mechanism exploration.

**Methods:**

A systematic search was conducted in the PubMed, Cochrane, Web of Science, and Embase (inception to January 2025) databases for preclinical studies on BMSC-EV intervention in osteoporosis models. A random-effects model was used to synthesize and analyze seven key parameters (BMD, BV/TV, Tb.N, Tb.Sp, Tb.Th, Ct.Th, and ultimate load-bearing capacity). Subgroup analysis was performed based on species (rats/mice), EVs engineering targets/methods, injection frequency, and treatment duration. The quality of the studies was assessed using SYRCLE’s risk of bias tool.

**Results:**

The meta-analysis of 10 studies (355 animals) showed that, compared to the control group, BMSC-EV treatment significantly increased BMD, BV/TV, Tb.N, Tb.Th, Ct.Th, and ultimate load-bearing capacity, while reducing Tb.Sp in the osteoporosis model. A publication bias was found in the summary analysis for Tb.N. However, sensitivity analysis confirmed that all summary results were relatively stable.

**Conclusions:**

Compared to the control group, BMSC-EV treatment demonstrated positive effects in increasing BMD, improving trabecular microstructure, cortical thickness, and biomechanical properties in the osteoporosis model. However, clinical translation still requires standardized EV characterization and preclinical safety assessments.

## Introduction

Osteoporosis is a common metabolic bone disease characterized by decreased bone mineral density (BMD) and disrupted bone microstructure, leading to increased bone fragility and elevated fracture risk [1]. In postmenopausal women, its incidence has significantly increased with the aging global population [2]. Due to the high prevalence of osteoporosis and its associated health burden, which affects over 200 million people worldwide, it has become a global public health issue [3]. Osteoporotic fractures, particularly hip and vertebral fractures, are linked to increased mortality and medical costs [4]. It has been reported that osteoporosis leads to more than 8.9 million fractures annually worldwide [5]. In the treatment of osteoporosis, recent years have seen some progress in clinical pharmacotherapy. As a disease of bone metabolism imbalance, current treatments include anti-resorptive drugs such as bisphosphonates, selective estrogen receptor modulators (SERMs), and RANKL inhibitors, as well as bone anabolic drugs such as teriparatide [6,7]. Previous clinical studies have shown that patients receiving anti-resorptive and bone anabolic therapies have a negative correlation between BMD levels and the subsequent risk of fractures [8,9]. However, the long-term use of these medications may result in side effects, such as delayed anabolic effects, drug resistance, and long-term safety concerns (e.g., bisphosphonate-related osteonecrosis of the jaw, atypical femoral fractures) [10,11]. These side effects have raised public concerns regarding the safety of these drugs, highlighting the urgent need for new treatment strategies that regulate bone metabolism while minimizing side effects [12].

Currently, extracellular vesicles (EVs) have attracted attention as a novel and effective intercellular communication mechanism [13]. EVs carry various biological signals and components, such as microRNAs, proteins, and lipids, enabling them to influence the function and biological activity of target cells in both physiological and pathological processes [14]. Their diverse effects on basic biological processes are mediated through various mechanisms, including the fusion of their membrane contents with the plasma membrane of recipient cells, the activation of cell surface receptors via bioactive lipids and proteins, and the delivery of effectors such as oncogenes, transcription factors, and non-coding regulatory RNAs [15,16]. Osteoblasts derived from bone marrow mesenchymal stem cells (BMSCs) play a key role in bone regeneration by secreting extracellular matrix (ECM) proteins, including type I collagen and other non-collagenous proteins [17,18]. BMSCs are considered promising candidates for bone regeneration due to their inherent osteogenic differentiation potential and paracrine activity. However, cell-based therapies face clinical translational barriers, including immune rejection, low post-transplantation survival rates, and ethical concerns [19].

Recent advances have highlighted the critical role of BMSC-derived extracellular vesicles (BMSC-EVs) as key mediators of intercellular communication in osteoporosis bone remodeling, including the regulation of osteoblast proliferation, differentiation, and apoptosis [20,21]. You et al. [22] found that BMSC-EVs isolated from femoral bone marrow of trauma patients significantly promoted the proliferation, differentiation, and alkaline phosphatase (ALP) activity of human osteoblasts *in vitro*. Furthermore, BMSC-EVs have been shown to improve bone loss in mouse models of osteoporosis, increasing bone mass and trabecular number [23]. Notably, BMSC-EVs can also enhance endothelial cell migration, proliferation, and tube formation, thereby promoting angiogenesis, which is critical for the vascular-bone coupling mechanism [24]. However, there is currently no evidence from clinical studies evaluating the effects of BMSC-EVs on bone mass and microstructural parameters in osteoporosis models. Current research evidence is primarily derived from animal models, with osteoporosis induction methods including ovariectomy (OVX), hormone induction, mechanical unloading, and genetic engineering approaches [25]. Regardless of whether the model is established through surgery, pharmacological agents, dietary manipulation, mechanical means, or genetic modifications, the primary objective remains the induction of bone loss characterized by reduced bone mass and disruption of bone microarchitecture. Therefore, the evaluation of the therapeutic efficacy of BMSC-EVs in osteoporosis models is primarily based on preclinical animal models, and they demonstrate promising therapeutic potential.

This systematic review and meta-analysis aim to consolidate preclinical data on the use of BMSC-EVs in treating osteoporosis animal models, assessing their potential impact and limitations on bone density, trabecular microstructure, and biomechanics. The findings will provide theoretical support for the future acceleration of BMSC-EV-based therapies for clinical translation in the treatment of osteoporosis.

## Materials and methods

### Systematic review

The study protocol was pre-established according to guidelines of the Preferred Reporting Items for Systematic Reviews and Meta-Analyses (PRISMA) checklist [26]. International Platform of Registered Systematic Review and Meta-analysis Protocols (IMPLASY) registration number (INPLASY202540080).

### Search strategy

Two independent authors conducted a comprehensive search of relevant literature through four public databases (PubMed, Cochrane Library, Embase, and Web of Science), with the search cutoff date set to January 30, 2025. The search strategy was constructed using both Medical Subject Headings (MeSH) and free-text terms (Emtree Terms), incorporating AND/OR combinations for database querying. A manual review of the reference lists from included studies was also performed to identify potentially relevant research. The search strategies for the four databases can be found in the supplementary materials. Discrepancies between the findings of two authors were discussed and resolved with input from a third author.

### Study selection criteria

Inclusion criteria:

1) Preclinical animal studies, which must include both intervention and control groups (e.g., blank control, placebo control);
2) Osteoporosis animal models of any species, such as rats, mice, etc., with model induction methods including but not limited to OVX, glucocorticoids (e.g., dexamethasone), and age-related osteoporosis models;
3) The intervention group must use BMSC-EVs, with no restrictions on the administration route;
4) The study must include at least one of the following bone metabolism-related indicators: bone mineral density (BMD), bone volume/total volume (BV/TV), trabecular number (Tb.N), trabecular separation (Tb.Sp), trabecular thickness (Tb.Th), cortical thickness (Ct.Th), and ultimate load-bearing capacity of the femur.

Exclusion criteria:

1) *In vitro* cell studies, human clinical trials, case reports, reviews, conference abstracts;
2) Studies using non-BMSC-EVs (e.g., EVs derived from adipose stem cells, umbilical stem cells) or EVs combined with other bioactive substances (e.g., BMP-2, VEGF);
3) Non-osteoporosis-related bone disease models (e.g., fracture healing models, bone tumor models);
4) Studies without a control group or using other active drugs as controls;
5) Studies where the full text cannot be accessed or data presentation is incomplete.

### Study selection

The study selection process was conducted in three stages: First, duplicate records were automatically removed using EndNote X20. Next, two independent authors performed an initial screening based on the titles/abstracts of the articles, and discrepancies in the screening results were resolved through discussions with a third author. In the third stage, full-text articles were retrieved and reviewed to further screen for studies that met the inclusion criteria. The screening process followed the PRISMA guidelines, and the results of the database searches and reasons for exclusions were documented in a standardized flowchart.

### Data extraction

A standardized electronic spreadsheet was used to collect general information from the included authors, year, animal characteristics (species, sample size, modeling methods), EVs characteristics (isolation, purification, and diameter), intervention protocols (EVs administration frequency, routes, and duration), and various outcome indicators (BMD, BV/TV, Tb.N, Tb.Sp, Tb.Th, Ct.Th, and ultimate load-bearing capacity). Two independent researchers used Origin software (version 2021) to extract numerical data independently from statistical graphs, presenting the data as mean ± standard deviation (mean ± SD). Discrepancies in the data extraction results were resolved through discussions with a third researcher. For missing standard deviations or unclear results, attempts were made to contact the corresponding authors to obtain the original data.

### Primary and secondary outcomes

Primary outcomes included BMD, BV/TV, Tb.Th, Tb.N, and Tb.Sp, which reflect bone density and bone strength indicators. Secondary outcomes focused on Ct.Th and femoral ultimate load. Additionally, for results with significant heterogeneity (*I*^2^ ≥ 50%), subgroup analyses were conducted based on species (rats/mice), EVs engineering targets/methods, injection frequency, and treatment duration.

### Risk of bas assessment

Two authors independently assessed the quality of the studies using the Systematic Review Centre for Laboratory Animal Experimentation (SYRCLE) risk of bias tool, which evaluates four areas [27]: randomization, allocation concealment, blinding, and outcome reporting. Discrepancies in the results were resolved through discussions with a third author. For each study, the risk of bias for specific criteria was recorded as “low”, “unclear” or “high” and the results were visualized using Review Manager (RevMan) 5.4.

### Statistical analysis

A random-effects model was applied to calculate the standardized mean differences (SMDs) with 95% confidence intervals for all outcomes. Heterogeneity was quantified using the *I*² statistic and *χ*² test. According to the Cochrane Handbook, the levels of heterogeneity are classified as follows: 0%−40% considered low, 30%−60% moderate, 50%−90% substantial, and 75%−100% considerable heterogeneity. Pre-specified subgroup analyses (species, engineering methods/targets, administration frequency, and duration) were performed when *I*² ≥ 50%. Sensitivity analysis was conducted to assess the stability of the results. Potential publication bias was evaluated using funnel plots and Egger’s regression test. All analyses were conducted using Review Manager (RevMan) 5.4 and Stata 17.0 with *p* < 0.05 considered statistically significant.

## Results

### Literature selection process

**Fig 1:**
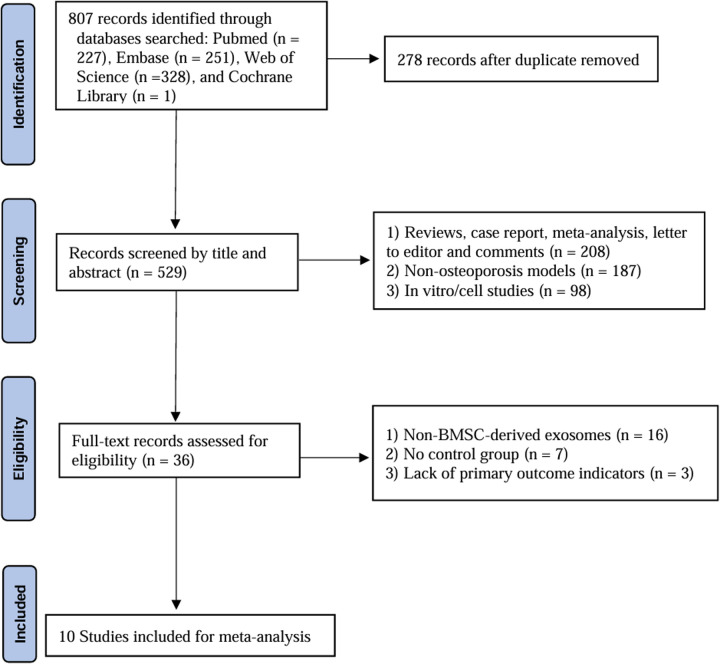
The PRISMA flowchart illustrates the study selection process.

A comprehensive search of four databases (PubMed, Embase, Web of Science, and Cochrane) yielded a total of 807 articles. After removing duplicates, the titles and abstracts were screened, resulting in the exclusion of 493 articles. Subsequently, 36 articles were reviewed in full text, and 26 were excluded for the following reasons: 16 studies used non-BMSC-derived EVs, 7 had no control group, and 3 lacked key outcome indicators. Ultimately, 10 studies [28–37] were included in this systematic review and meta-analysis. The PRISMA flowchart for the study selection process is shown in Fig 1.

### Study characteristics

#### Characteristics of animal models.

The 10 included studies were published within the past five years (2020–2024), indicating the growing attention on BMSC-EVs for the treatment of osteoporosis in recent years. All studies were conducted in China. Regarding the basic characteristics of the animal models, one study did not specify the species used, while 4 studies employed Sprague Dawley (SD) rats, and 5 studies used C57 mice as modeling subjects. Eight studies used female animals, and two studies used male animals. The age range of the animals was from 6 weeks to 6 months. Only 4 studies reported the body weight of animals. For the modeling methods, 8 studies induced osteoporosis through ovariectomy, 1 study used hindlimb unloading (HU)-induced osteoporosis, and 1 study did not report the modeling details. Table 1 describes the main characteristics of the animal models.

**Table 1:** Main characteristics of studies included.

Author	Year	Country	Specie	Gender	Age	Weight	Number	Model of osteoporosis
Gui et al [28]	2024	China	C57BL/6 mice	female	8-week-old	Not description	50	Ovariectomized
Huang et al [29]	2021	China	Sprague Dawley (SD) rats	female	10-week-old,	230 - 250 g	40	Ovariectomized
Li et al [30]	2021	China	Sprague Dawley (SD) rats	female	8-week-old	294 ± 11 g	40	Ovariectomized
Li et al [31]	2023	China	Not description	female	6-week-old	Not description	24	Ovariectomized
Lu et al [32]	2020	China	C57BL/6J mice	male	3-month-old	Not description	15	Not description
Qi et al [33]	2023	China	Sprague Dawley (SD) rats	female	10 weeks old	230–250 g	18	Ovariectomized
Qiu et al [34]	2020	China	Sprague Dawley (SD) rats	female	12 weeks old	280-300 g	66	Ovariectomized
Wang et al [35]	2023	China	C57BL/6 mice	female	Not description	Not description	42	Ovariectomized
Xiao et al [36]	2021	China	C57BL/6J mice	male	6-month-old	Not description	20	Suspended from their hindlimbs for a period of 28 days
Yang et al [37]	2022	China	C57BL/6J mice	female	8-week-old	Not description	40	Ovariectomized

#### Characteristics of BMSC-EVs intervention.

**Table 2** summarizes the intervention characteristics of BMSC-EVs. In the included studies, the sources of BMSCs were from mice (N = 5), humans (N = 3), and rats (N = 2). The isolation methods for BMSC-EVs included ultracentrifugation (N = 7) and precipitation kits (N = 2), while one study did not report the purification details. Purification approaches involved filtration through a 0.22/0.45 μm membrane (N = 6) and PBS-washed precipitation (N = 1); three studies did not specify purification procedures. The particle size range of EVs was 30–500 nm. Regarding the EVs intervention characteristics, 8 studies used intravenous injection, 1 study used intraperitoneal injection, and 1 study did not describe the administration route. The dosage for 5 studies was 100 μg, while the remaining 5 studies had varying dosages. The administration frequencies included once a week (N = 5), twice a week (N = 3), three times a week (N = 1), and once daily (N = 1). Treatment durations were 2 weeks (N = 1), 4 weeks (N = 5), 2 months (N = 3), and 3 months (N = 1).

**Table 2:** Characteristics of EVs and therapeutic method.

Author	Year	Characteristics of EVs							Therapeutic methods			
		Isolation and purification				EV characterization						
		Source	Cell culture	Isolation	Purification	TEM	Marker	Diameter (nm)	Route	Dose	Time	Duration
Gui et al [28]	2024	mBMSCs	BMSCs were treated with staurosporine (0.5 µM) for 6h	Ultracentrifugation	Not description	Cup-shaped morphology	PKH67	220-396 nm	intravenously	10 mg/kg	once a week	4 weeks
Huang et al [29]	2021	rBMSCs	Cultivate to P2-P4	Ultracentrifugation	Filtered through a 0.22 μm filter	Cup- or sphere-like morphology	CD9, CD63, and CD81	40-120 nm)	intravenously	100 μg	once a week	2 months
Li et al [30]	2021	hBMSCs	Cultivate	Polymer precipitation kits	Not description	Not description	Alixs, CD63, and CD81	100-150nm	intravenously	1 × 10^13^/mL	once a week	1 month
Li et al [31]	2023	hBMSCs	Cultivate	Not description	Not description	Not description	Not description	Not description	intravenously	/	once a week	4 weeks
Lu et al [32]	2020	mBMSCs	Cultivate	Ultracentrifugation	Filtered through a 0.22 μm filter	Round shape	Syntenin 1, and TSG101	30-150nm	/	100 μg	twice a week	2 months
Qi et al [33]	2023	rBMSCs	Cultivate to P3	Ultracentrifugation	Filtered through a 0.22 μm filter	Hollow spherical microvesicles	CD63, CD81, and TSG101	50-120 nm	intravenously	100 μL	once a week	2 months
Qiu et al [34]	2020	rBMSCs	Cultivate to P3	ExoEasy Maxi Kit	Filtered through a 0.45 μm filter	Low-density electrons in the vesicles	CD63 and CD9	30-100 nm	intravenously	100 μg	once a days	2 weeks
Wang et al [35]	2023	mBMSCs	Cultivate	Ultracentrifugation	Filtered through a 0.22 μm filter	Cup-shaped morphology	CD9, CD63, and CD81	100 nm	intravenously	100 μg	twice a week	3 months
Xiao et al [36]	2021	mBMSCs	Cultivate to 80–90%	Ultracentrifugation	Filtered through a 0.22 μm filter	Round shape	CD63 and TSG101	40-260 nm	intravenously	0.1 mL per injection	twice a week	4 weeks
Yang et al [37]	2022	mBMSCs	Cultivate to 50–60%	Ultracentrifugation	The pellet was washed with PBS	Cup-shaped morphology	Alix, CD63, TSG101, and CD81	500nm	intraperitoneally	100 μg	thrice a week	4 weeks

### Risk of bias assessment

**Fig 2:**
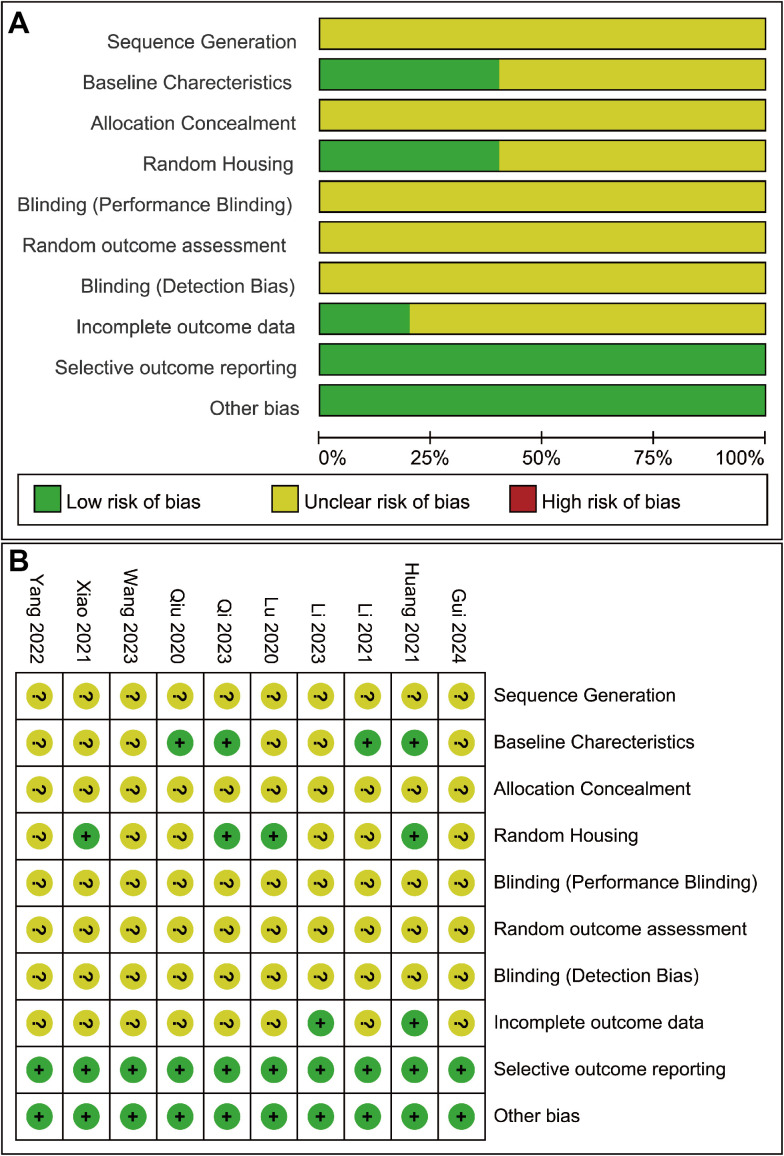
Risk of bias assessment of the 10 included studies based on SYRCLE’s ROB tool. **(A)** Risk of bias graph; **(B)** Risk of bias summary.

The risk of bias assessment results show that all the included studies had unclear risk of bias. Specifically, in the five domains, including sequence generation, allocation concealment, performance blinding, random outcome assessment, and detection blinding, all studies failed to disclose details, resulting in uncertain risk of bias. Four studies provided detailed reports on the animal model characteristics, including number, gender, species, age, weight, and modeling method. Additionally, four studies described the randomization of animal placement. The bias risk assessment for each study and the summarized results are shown in Fig 2A and 2B.

### Outcomes

#### BMD.

**Fig 3:**
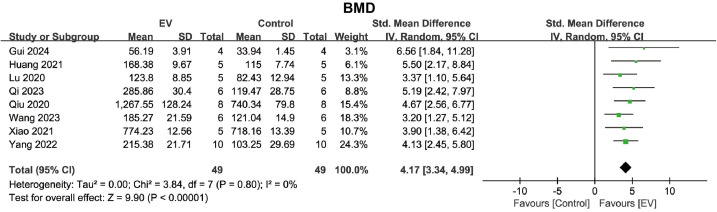
Forest plot showing the effect of BMSC-EVs on BMD in the osteoporosis model. Data are presented as standardized mean differences (SMD) with 95% confidence intervals (CI).

Data from 8 studies met the criteria for inclusion in the meta-analysis. Compared to the control group, BMSC-EVs significantly increased the BMD in the osteoporosis model (SMD = 4.17, 95% CI: 3.34 to 4.99, *p* < 0.00001) (Fig 3). The heterogeneity test showed no significant heterogeneity in the BMD results (*I*² = 0%, *p* = 0.80), so no further subgroup analysis was performed. These findings indicated a positive effect of BMSC-EVs in enhancing bone mineral density in the osteoporosis model.

#### Bone mass.

**Fig 4:**
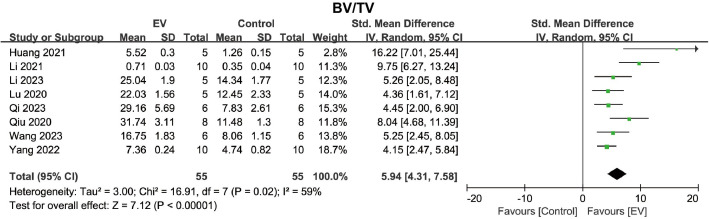
Forest plot showing the effect of BMSC-EVs on BV/TV in the osteoporosis model. Data are presented as standardized mean differences (SMD) with 95% confidence intervals (CI).

BV/TV directly reflects changes in bone mass. Eight studies evaluated the changes in bone BV/TV in the osteoporosis model following BMSC-EVs treatment. The pooled analysis showed that BMSC-EVs significantly increased the BV/TV in the osteoporosis model (SMD = 5.94, 95% CI: 4.31 to 7.58, *p* < 0.00001) (Fig 4). Heterogeneity analysis revealed significant heterogeneity (*I*² = 59%, *p* = 0.02). Subgroup analysis indicated that BMSC-EVs significantly improved BV/TV across different animal types (rats or C57 mice), isolation methods (ultracentrifugation), purification approaches (filtration through a 0.22/0.45 μm membrane), EV size (small EVs), intervention frequency (once weekly), and treatment durations (≤4 weeks or >4 weeks) (S1–S6 Figs). However, due to significant heterogeneity in some subgroup analyses, caution should be exercised when interpreting these results. Notably, the heterogeneity in BV/TV was not resolved through subgroup analysis, suggesting that none of the subgroups significantly impacted the heterogeneity. These results indicate that BMSC-EVs are associated with an increase in bone mass in the osteoporosis model.

#### Trabecular structural parameters.

**Fig 5:**
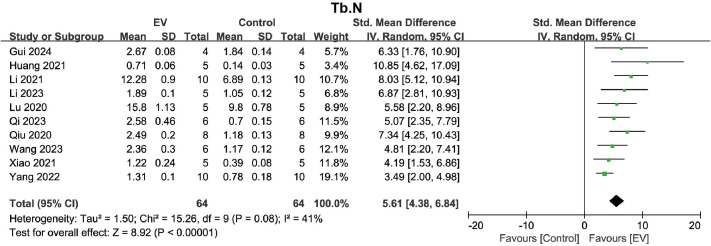
Forest plot indicating the effect of BMSC-EVs on Tb.N in the osteoporosis model. Data are presented as standardized mean differences (SMD) with 95% confidence intervals (CI).

**Fig 6:**
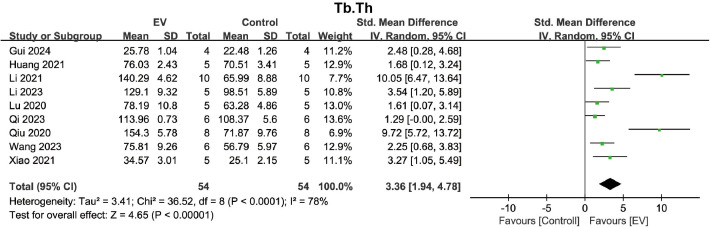
Forest plot indicating the effect of BMSC-EVs on Tb.Th in the osteoporosis model. Data are presented as standardized mean differences (SMD) with 95% confidence intervals (CI).

The bone quality of trabeculae is closely related to its microstructure, with key indicators including Tb.N, Tb.Th, and Tb.Sp. Ten studies reported the Tb.N after treatment in the osteoporosis model. The pooled analysis showed that, compared to the control group, BMSC-EVs significantly increased the Tb.N in the osteoporosis model (SMD = 5.61, 95% CI: 4.38 to 6.84, *p* < 0.00001) (Fig 5). Due to moderate heterogeneity (*I*² = 41%, *p* = 0.08), no further subgroup analysis was conducted. A pooled analysis of 9 studies showed that BMSC-EVs significantly increased the Tb.Th in the osteoporosis model (SMD = 3.36, 95% CI: 1.94 to 4.78, *p* < 0.00001) (Fig 6). Due to significant heterogeneity (*I*² = 78%, *p* < 0.0001), subgroup analysis was conducted to identify significant factors influencing heterogeneity. Subgroup analyses based on six different grouping strategies demonstrated that BMSC-EVs improved Tb.Th in various subgroups, including the rats subgroup, C57 mice subgroup, ultracentrifugation subgroup, filter-based purification subgroup, small EVs subgroup, once-weekly intervention subgroup, ≤ 4 weeks subgroup, and >4 weeks subgroup (S7–S12 Figs). However, none of the subgroups were significant factors influencing heterogeneity.

**Fig 7:**
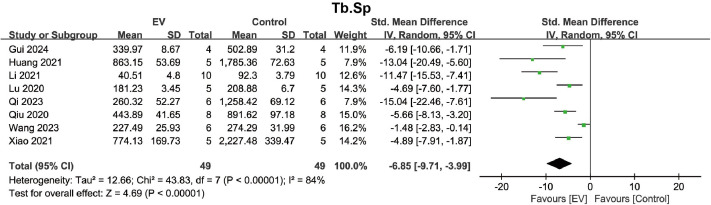
Forest plot showing the effect of BMSC-EVs on Tb.Sp in the osteoporosis model. Data are presented as standardized mean differences (SMD) with 95% confidence intervals (CI).

For Tb.Sp, a pooled analysis of 8 studies indicated that BMSC-EVs significantly reduced the Tb.Sp in the osteoporosis model (SMD = −6.85, 95% CI: −9.71 to −3.99, *p* < 0.00001) (Fig 7). Due to significant heterogeneity (*I*² = 84%, *p* < 0.00001), subgroup analysis was performed to identify significant factors contributing to the heterogeneity. Subgroup analyses based on six different stratification methods showed that BMSC-EVs consistently reduced Tb.Sp (S13–S18 Figs). However, none of the subgroups were identified as significant factors influencing heterogeneity. These results suggest that BMSC-EVs play a positive role in improving the trabecular spatial structure of the osteoporosis model, including increasing Tb.N and Tb.Th, and decreasing Tb.Sp.

#### Cortical bone parameters.

**Fig 8:**
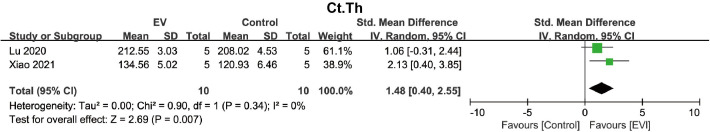
Forest plot demonstrating the effect of BMSC-EVs on Ct.Th in the osteoporosis model. Data are presented as standardized mean differences (SMD) with 95% confidence intervals (CI).

Two studies reported the Ct.Th in animal models after treatment. The analysis showed that, compared to the control group, BMSC-EVs significantly increased the Ct.Th in the osteoporosis model (SMD = 1.48, 95% CI: 0.40 to 2.55, *p* = 0.007) (Fig 8). However, due to the small number of studies included in the analysis, caution should be exercised when interpreting this result.

#### Ultimate load-bearing capacity of femur.

**Fig 9:**
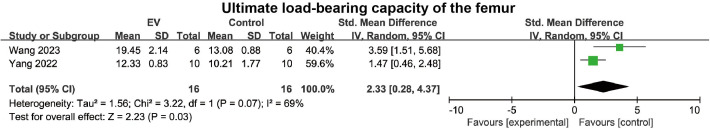
Forest plot showing the effect of BMSC-EVs on the ultimate load-bearing capacity of the femur in the osteoporosis model. Data are presented as standardized mean differences (SMD) with 95% confidence intervals (CI).

Additionally, we analyzed the impact of BMSC-EVs treatment on the biomechanical indicator of the femur, the ultimate load-bearing capacity. The pooled results showed that, compared to the control group, BMSC-EVs significantly increased the ultimate load-bearing capacity of the femur in the osteoporosis model (SMD = 2.33, 95% CI: 0.28 to 4.37, *p* = 0.03) (Fig 9). However, due to the small number of studies included in the analysis, caution should be exercised when interpreting this result.

### Sensitivity analysis

**Fig 10:**
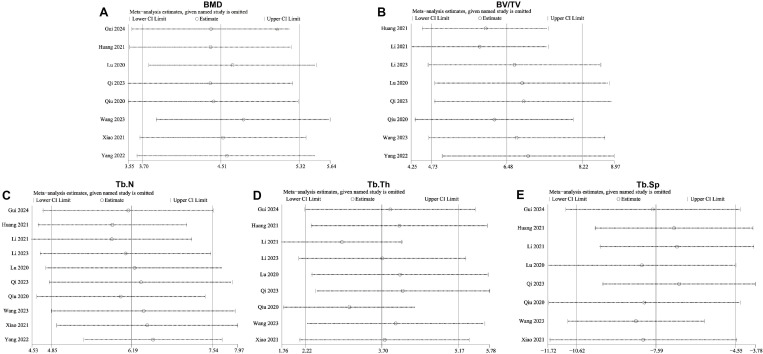
Sensitivity analysis results for various outcome measures. **(A)** Bone mineral density (BMD); (B) bone volume fraction (BV/TV); (C) trabecular number (Tb. **N)**; (D) trabecular thickness (Tb. Th); (E) trabecular separation/marrow thickness (Tb. Sp).

We conducted a sensitivity analysis for the five primary outcome indicators: BMD, BV/TV, Tb.Th, Tb.N, and Tb.Sp, to assess the stability of the pooled analysis results. The sensitivity analysis for BMD showed that removing any single study did not significantly alter the combined effect size, indicating the stability of the pooled BMD results (Fig 10A). Furthermore, sensitivity analyses for BV/TV, Tb.N, Tb.Th, and Tb.Sp all showed that the differences between results with and without each study were not statistically significant, suggesting that the study results were stable (Fig 10B–10E).

### Publication bias

**Fig 11:**
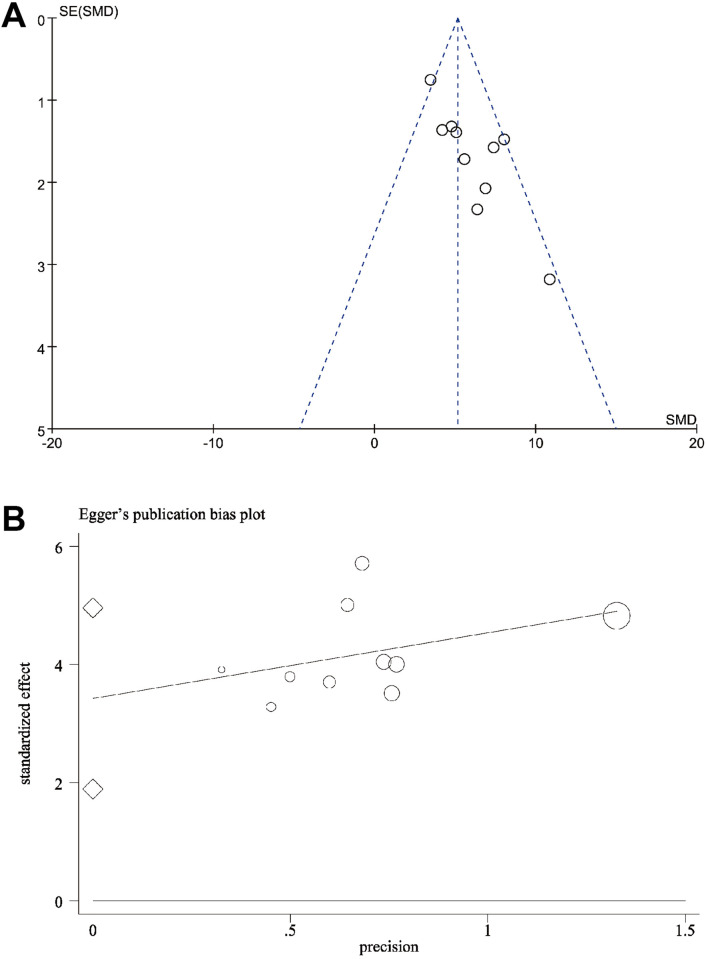
Publication bias assessment of Tb. N. **(A)** Funnel plot with pseudo-95% confidence limits; **(B)** Publication bias plot of Egger’s test.

Additionally, we assessed publication bias for the 10 studies reporting Tb.N. The funnel plot showed asymmetry, and further Egger’s test confirmed the presence of publication bias (*t* = 5.15, *p* = 0.001) (Fig 11A and 11B).

## Discussion

In this systematic review and meta-analysis, we comprehensively evaluated the efficacy of BMSC-EVs in osteoporosis animal models. The aggregated analysis results indicate that BMSC-EV treatment significantly increased BMD and bone volume (BV/TV), improved trabecular microstructure (increased BV/TV, Tb.N, Tb.Th; decreased Tb.Sp), cortical thickness (Ct.Th), and biomechanical strength, suggesting that BMSC-EVs have a positive impact on bone density and microstructural parameters in osteoporosis models. Further subgroup analysis revealed that animal species, engineering methods/targets, EV intervention frequency, and treatment duration did not affect the improvement of bone mass and trabecular microstructure in osteoporosis models by BMSC-EVs. Given the adverse reactions and side effects of traditional drugs, BMSC-EVs may offer a viable strategy for the clinical treatment of osteoporosis.

### BMSC-EVs Enhanced BMD and bone mass

From a microscopic perspective, osteoblasts mainly originate from BMSCs with strong osteogenic differentiation potential and play a critical role in bone metabolism [38]. Numerous previous studies have demonstrated the promoting effects of BMSC-EVs on bone repair both *in vitro* and *in vivo*. Zhao et al. [39] found that mandibular BMSC-EVs may promote osteogenic differentiation *in vitro* through the TLR4/NF-κB signaling pathway, providing potential insights for osteoporosis treatment. Another study showed that BMSC-EVs could deliver MiR-16-5p, inhibiting Axin2, thereby promoting osteogenic differentiation [40]. Li et al. [41] found that apoptotic BMSC-EVs effectively promoted the proliferation, migration, and osteogenic differentiation of BMSCs *in vitro*. In osteoporosis mouse models, apoptotic BMSC-EVs alleviated bone loss and promoted bone repair, as indicated by increased BV/TV. Additionally, Wang et al. [42] also discovered that EVs derived from young osteocytes could significantly increase BV/TV and bone mass in aged mouse models. In this meta-analysis, we also found that BMSC-EV treatment significantly improved BMD and BV/TV in osteoporosis animal models, suggesting that BMSC-EVs may improve osteoporosis by increasing BMD and bone mass.

In clinical treatment, BMD is often used as a key measure of the efficacy of drug interventions [43]. Several previous meta-regression analyses have shown that greater improvements in BMD are closely associated with significant reductions in vertebral and hip fractures, indicating that improvements in BMD levels during drug intervention trials may serve as a surrogate endpoint for osteoporotic fractures [44,45]. The significant improvements in BMD and BV/TV brought about by BMSC-EVs suggest their potential as a cell-free therapy for treating osteoporotic fractures. It is noteworthy that BMD often rapidly declines after discontinuation of most medications, leading to an increased risk of multiple vertebral fractures [46]. A study on women with low bone mass found that a single infusion of zoledronic acid could maintain BMD levels for up to 5 years [47]. However, current evidence indicates that the duration for which BMSC-EVs can maintain BMD levels *in vivo* remains uncertain and is limited to animal models. Therefore, future research should focus on the long-term effectiveness of BMSC-EVs in maintaining BMD *in vivo*, which is crucial for their clinical translation.

### Restoration of trabecular microarchitecture

BMSC-EVs significantly improved trabecular microstructure parameters, with notable increases in Tb.N (SMD = 5.61) and Tb.Th (SMD = 3.36), while simultaneously reducing Tb.Sp (SMD = −6.85). These effects may stem from the dual action of BMSC-EVs in promoting osteogenesis and inhibiting osteoclast activity, thereby improving the bone metabolic imbalance associated with osteoporosis. However, there is currently insufficient *in vivo* evidence (such as immunohistochemical quantitative indicators) to analyze the potential effects of BMSC-EVs on osteoblasts and osteoclasts. Consistent with the trabecular microstructure results, Zhang et al. [48] found that microRNA-935-modified BMSC-EVs significantly increased Tb.N and Tb.Th, and reduced Tb.Sp in an osteoporotic rat model, demonstrating the improvement of trabecular parameters by BMSC-EVs. Another study indicated that BMSC-EVs significantly enhanced osteogenic activity and bone mass in an osteoporosis model of mouse, improving trabecular microstructure parameters and offering new insights for the treatment of osteoporosis [49].

Although the analysis results demonstrate the promising potential of BMSC-EVs for osteoporosis treatment, sensitivity analysis reveals the stability of the findings. However, significant heterogeneity remains in the summary analyses of BV/TV (*I*^2^ = 59%), Tb.Th (*I*^2^ = 78%), and Tb.Sp (*I*^2^ = 84%). This heterogeneity cannot be explained by subgroup analysis based on animal species, EV engineering methods, or intervention frequency and duration, and may reflect the influence of other unmeasurable variables, such as EV preparation processes, storage conditions, and factors that alter the biological activity of EVs. Therefore, for the clinical translation of BMSC-EVs, standardization of EV preparation and intervention, along with stringent safety evaluations, is essential.

### EV characteristics and significant heterogeneity

EVs exhibit a broad size range, and the sizes of different types of EVs vary inconsistently. According to the latest MISEV2023 guidelines [50], EVs with diameters less than 200 nm are generally classified as small EVs, although a consensus on the precise threshold has yet to be reached. Based on this classification, we conducted subgroup analyses of BV/TV, Tb.Th, and Tb.Sp for small EVs (<200 nm) and large EVs (≥200 nm). The results suggest that small EVs may play a beneficial role in increasing BV/TV and Tb.Th and in reducing Tb.Sp. Notably, small and large EVs exhibit distinct biodistribution and accumulation patterns in rodent models following administration [51]. Specifically, small EVs are initially detected in the liver and kidneys, while large EVs appear first in the lungs. Furthermore, compared with large apoptotic EVs (>1000 nm), small apoptotic EVs (<1000 nm) derived from MSCs more effectively induce macrophage polarization toward the M2 anti-inflammatory phenotype and promote wound healing in diabetic mice [52]. These findings provide evidence supporting the role of EV size in modulating osteoimmunological processes related to osteoporosis.

Subgroup analysis based on different EV isolation methods (ultracentrifugation or precipitation kits) indicated that the ultracentrifugation subgroup may enhance BV/TV and Tb.Th while reducing Tb.Sp in the models. Previous studies have shown that conventional isolation techniques such as ultracentrifugation and filtration may cause biomolecular and membrane damage, potentially triggering immune responses [53]. Additionally, both ultracentrifugation and precipitation kits can lead to substantial co-precipitation of protein contaminants [54], which may interfere with therapeutic efficacy. These factors may contribute to the significant heterogeneity observed in the analysis of Tb.Sp reduction within the ultracentrifugation subgroup. In contrast, tangential flow filtration achieves reduced EV damage by maintaining controlled shear rates [55]. However, none of the ten included studies employed this isolation method. Future research should prioritize standardized protocols for EV isolation and purification to generate higher-quality evidence of therapeutic outcomes.

Additionally, different routes of administration, dosages, and frequencies may influence the biodistribution, accumulation, and metabolism of EVs in vivo. In a mouse model study involving EV intervention, intravenous injection led to primary accumulation in the liver, intraperitoneal injection resulted in significantly increased accumulation in the gastrointestinal tract, and subcutaneous injection showed the lowest liver accumulation. Regarding dosage, both low and moderate doses (<1.0 × 10^10^ particles/g) predominantly accumulated in the liver, whereas high doses (1.5 × 10^10^ particles/g) resulted in reduced hepatic accumulation [56]. Among the ten included BMSC-EV intervention studies, only one employed intraperitoneal injection. Hematoxylin-eosin staining and biophotonic imaging analyses revealed no apparent systemic toxicity, with EV-loaded nanoparticles mostly cleared by the liver and no reported gastrointestinal accumulation [37]. Eight studies used intravenous injection, and two of these reported biosafety data, indicating no observable organ toxicity and no impairment of liver or kidney function [28,29]. Further studies are needed to investigate the metabolic and distributional differences as well as the underlying mechanisms associated with various administration routes, which are crucial for achieving consistent therapeutic efficacy and safety.

EVs derived from various cell types within the osteoimmune microenvironment play a critical role in regulating bone homeostasis, including those from osteoclasts, osteoblasts, and macrophages. Osteoclasts are key contributors to the development and progression of osteoporosis. A previous study demonstrated that EVs derived from osteoclasts promoted the osteogenic differentiation of MSCs *in vitro* by targeting *ARHGAP1*, a negative regulator of osteogenesis, and exhibited strong bone regenerative capacity in a mouse calvarial defect model [57]. Li et al. [58] confirmed that osteoclast-derived exosomal miR-214-3p enhances bone formation in aged OVX mice. The effects of osteoblast-derived EVs are more complex. Davies et al. [59] found that EVs from osteoblasts induced osteogenic mineralization in MSCs, marked by the upregulation of ALP and BMP-2. However, EVs isolated from osteoblasts of osteoporosis patients negatively regulated MSC osteogenic differentiation [60]. Moreover, macrophages modulate bone homeostasis through the secretion of cytokines and EVs. EVs from M1 macrophages, enriched in miRNA-155, exacerbate bone loss in osteoporosis models by downregulating DUSP1 and activating the JNK signaling pathway [61]. Another study showed that M1 macrophage-derived exosomes promoted the osteogenic differentiation of BMSCs during the early stages of inflammation via microRNA-21a-5p [62]. In summary, beyond BMSCs, multiple cell types can influence the osteoporotic microenvironment through EV secretion, yet the diversity and often contradictory effects of these regulatory mechanisms require further mechanistic investigation.

Currently, common animal models for osteoporosis include OVX-induced models, dexamethasone-induced models, and mechanical unloading models. Among the ten studies included in our analysis, eight employed OVX-induced osteoporosis models. Mechanistically, ovariectomy leads to estrogen deficiency, which in turn results in bone loss, manifesting as reduced bone mass and strength [25]. However, due to the inability of rats to achieve full skeletal maturity, OVX models using animals such as rabbits [63], Cynomolgus monkeys [64], and sheep [65] are increasingly being adopted. It is noteworthy that even when using OVX to induce osteoporosis, the timeline of bone loss differs between rats and mice [66,67], which may lead to variability in the therapeutic efficacy of BMSC-EVs. In dexamethasone-induced osteoporosis models, increased bone resorption and disruption of bone microarchitecture are the primary pathological features. In rat models, this is characterized by discontinuous and thin trabecular bone as well as irregularly eroded endosteal surfaces [68]. As for mechanical unloading models, the absence of mechanical stimuli may lead to elevated secretion of inflammatory cytokines and enhanced bone resorption, ultimately resulting in disuse osteoporosis [69]. However, the specific mechanisms remain to be fully elucidated. Due to the limited number of studies available, we were unable to draw definitive conclusions or provide detailed interpretations regarding the influence of non-OVX modeling methods on the efficacy of EVs.

## Limitations

This study has several limitations. First, all the included studies were conducted in China, which may introduce regional risk biases in the osteoporosis models and BMSC-EV preparation protocols. Second, incomplete reporting of key methodological details (such as EV storage conditions and administration time) hindered the resolution of significant heterogeneity. Third, the small sample sizes for cortical bone thickness (N = 2) and biomechanical analysis (N = 3) limited the statistical power, and further validation in larger samples is needed. Finally, the lack of safety data and immunogenicity assessments following BMSC-EV interventions emphasizes the need for standardized EV preparation and intervention protocols.

## Conclusions

In conclusion, this systematic review and meta-analysis highlights the potential therapeutic value of BMSC-EVs for osteoporosis animal models, including improvements in BMD, bone mass, trabecular microstructure parameters, and femoral ultimate load. However, due to the presence of heterogeneity, these results should be interpreted with caution. Future studies should further explore their clinical translational value based on standardized intervention protocols and comprehensive safety evaluations.

## Supporting information

S1 Fig Subgroup analysis showing the BV/TV results based on animal species (with their 95% confidence intervals). (DOCX)

S2 Fig Subgroup analysis showing the BV/TV results based on EVs isolation methods (with their 95% confidence intervals). (DOCX)

S3 Fig Subgroup analysis showing the BV/TV results based on EVs purification methods (with their 95% confidence intervals). (DOCX)

S4 Fig Subgroup analysis showing the BV/TV results based on EVs sizes (with their 95% confidence intervals). (DOCX)

S5 Fig Subgroup analysis showing the BV/TV results based on BMSC-EVs injection frequency (with their 95% confidence intervals). (DOCX)

S6 Fig Subgroup analysis showing the BV/TV results based on BMSC-EVs treatment duration (with their 95% confidence intervals). (DOCX)

S7 Fig Subgroup analysis showing the Tb.Th results based on animal species (with their 95% confidence intervals). (DOCX)

S8 Fig Subgroup analysis showing the Tb.Th results based on EVs isolation methods (with their 95% confidence intervals). (DOCX)

S9 Fig Subgroup analysis showing the Tb.Th results based on EVs purification methods (with their 95% confidence intervals). (DOCX)

S10 Fig Subgroup analysis showing the Tb.Th results based on EVs sizes (with their 95% confidence intervals). (DOCX)

S11 Fig Subgroup analysis showing the Tb.Th results based on BMSC-EVs injection frequency (with their 95% confidence intervals). (DOCX)

S12 Fig Subgroup analysis showing the Tb.Th results based on BMSC-EVs treatment duration (with their 95% confidence intervals). (DOCX)

S13 Fig Subgroup analysis showing the Tb. Sp results based on animal species (with their 95% confidence intervals).
(DOCX)

S14 Fig Subgroup analysis showing the Tb.Sp results based on EVs isolation methods (with their 95% confidence intervals). (DOCX)

S15 Fig Subgroup analysis showing the Tb. Sp results based on EVs purification methods (with their 95% confidence intervals).
(DOCX)

S16 Fig Subgroup analysis showing the Tb.Sp results based on EVs sizes (with their 95% confidence intervals). (DOCX)

S17 Fig Subgroup analysis showing the Tb.Sp results based on BMSC-EVs injection frequency (with their 95% confidence intervals). (DOCX)

S18 Fig Subgroup analysis showing the Tb.Sp results based on BMSC-EVs treatment duration (with their 95% confidence intervals). (DOCX)

## Abbreviations:

ALP: Alkaline phosphatase
BMD: bone mineral density
BMSC: bone marrow mesenchymal stem cell
BMSC-EVs: bone marrow mesenchymal stem cell-derived extracellular vesicles
BV/TV: bone volume/total volume
Ct.Th: cortical thickness
DXA: dual-energy X-ray absorptiometry
ECM: extracellular matrix
EVs: extracellular vesicles
HU: hindlimb unloading
MeSH: medical subject headings
OVX: ovariectomy
PRISMA: preferred reporting items for systematic reviews and meta-analyses
SD: sprague dawley
SERMs: selective estrogen receptor modulators
SMD: standardized mean difference
SYRCLE: systematic review centre for laboratory animal experimentation
Tb.N: trabecular number
Tb.Sp: trabecular separation
Tb.Th: trabecular thickness
