# Supplementary material for: The effect of bone marrow mesenchymal stem cell-derived extracellular vesicles on bone mineral density and microstructure in osteoporosis: A systematic review and meta-analysis of preclinical studies
Source: PLoS One. 2025 Jun 30;20(6):e0327011. doi: 10.1371/journal.pone.0327011 (PMC12208419; doi:10.1371/journal.pone.0327011)
Supplement: S12 Fig — (DOCX) [file pone.0327011.s012.docx]

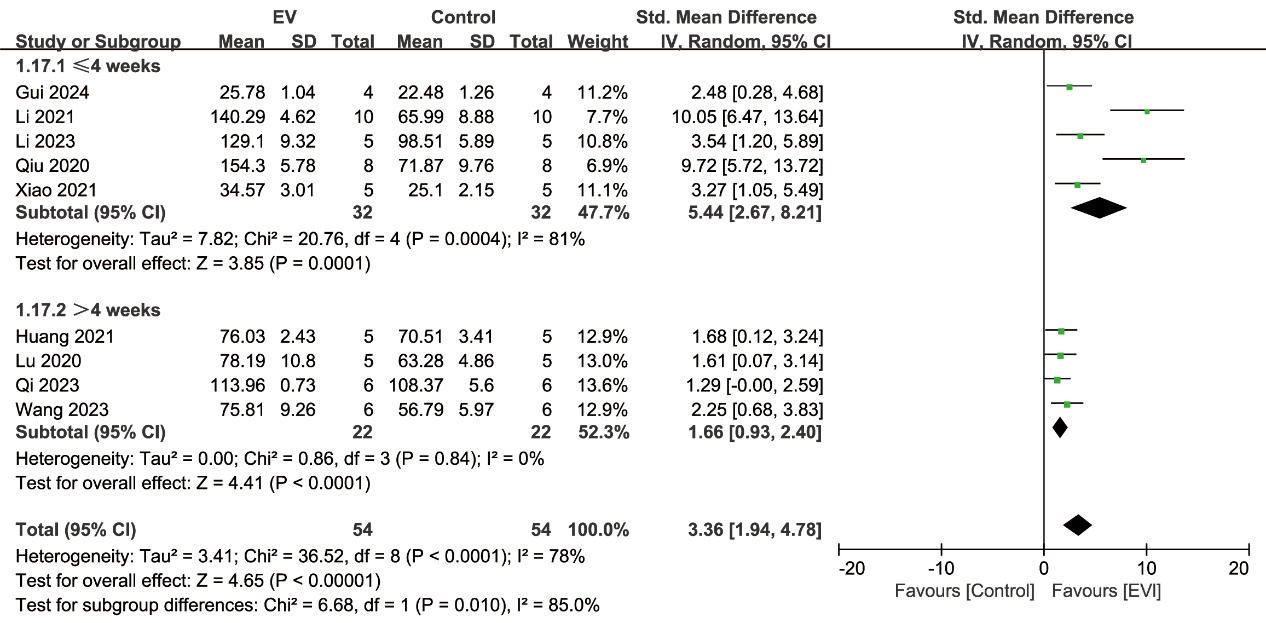


**S12 Fig. Subgroup analysis showing the Tb.Th results based on BMSC-EVs treatment duration (with their 95% confidence intervals).**
