# Supplementary material for: The effect of bone marrow mesenchymal stem cell-derived extracellular vesicles on bone mineral density and microstructure in osteoporosis: A systematic review and meta-analysis of preclinical studies
Source: PLoS One. 2025 Jun 30;20(6):e0327011. doi: 10.1371/journal.pone.0327011 (PMC12208419; doi:10.1371/journal.pone.0327011)
Supplement: Attachment — Submitted filename: Point-by-point response to the comments.docx [file pone.0327011.s020.docx]

**Point-by-point response to the editor and reviewers’ comments**

**#Reviewer 1**

In this review Authors provided a valuable and timely systematic evidence on the therapeutic relevance of BMSCs-derived EVs.

The study is interesting and well organized and the findings are clearly presented. In any case, the manuscript requires the following enhancements:

1. The introduction does not sufficiently cover the diversity of preclinical models used to simulate osteoporosis. Including descriptions of OVX, glucocorticoid-induced, and mechanical unloading models would improve the readers’ understanding of the model-dependent outcomes and their translational implications.

**Response**:

Thank you so much for providing us with the opportunity to revise our manuscript and for your constructive suggestions. As you rightly pointed out, the original "Introduction" section did not comprehensively reflect the diversity of osteoporosis animal models, including OVX, glucocorticoid-induced, mechanical unloading, and genetically engineered models, which may have affected the clarity and coherence for readers. In response, we have added a brief overview of these modeling approaches in the revised "Introduction." Furthermore, we have elaborated in the "Discussion" section on the principles and mechanisms underlying several commonly used osteoporosis models, while emphasizing that the mechanisms of these models remain to be fully elucidated. We also acknowledged the limitations of the studies included and provided perspectives for future research. The specific revisions and additions are as follows:

Current research evidence is primarily derived from animal models, with osteoporosis induction methods including ovariectomy (OVX), hormone induction, mechanical unloading, and genetic engineering approaches (1). Regardless of whether the model is established through surgery, pharmacological agents, dietary manipulation, mechanical means, or genetic modifications, the primary objective remains the induction of bone loss characterized by reduced bone mass and disruption of bone microarchitecture. Therefore, the evaluation of the therapeutic efficacy of BMSC-EVs in osteoporosis models is primarily based on preclinical animal models, and they demonstrate promising therapeutic potential.

Currently, common animal models for osteoporosis include OVX-induced models, dexamethasone-induced models, and mechanical unloading models. Among the ten studies included in our analysis, eight employed OVX-induced osteoporosis models. Mechanistically, ovariectomy leads to estrogen deficiency, which in turn results in bone loss, manifesting as reduced bone mass and strength (1). However, due to the inability of rats to achieve full skeletal maturity, OVX models using animals such as rabbits (2), Cynomolgus monkeys (3), and sheep (4) are increasingly being adopted. It is noteworthy that even when using OVX to induce osteoporosis, the timeline of bone loss differs between rats and mice (5, 6), which may lead to variability in the therapeutic efficacy of BMSC-EVs. In dexamethasone-induced osteoporosis models, increased bone resorption and disruption of bone microarchitecture are the primary pathological features. In rat models, this is characterized by discontinuous and thin trabecular bone as well as irregularly eroded endosteal surfaces (7). As for mechanical unloading models, the absence of mechanical stimuli may lead to elevated secretion of inflammatory cytokines and enhanced bone resorption, ultimately resulting in disuse osteoporosis (8). However, the specific mechanisms remain to be fully elucidated. Due to the limited number of studies available, we were unable to draw definitive conclusions or provide detailed interpretations regarding the influence of non-OVX modeling methods on the efficacy of EVs.

2. The current review focuses exclusively on BMSC-EVs, yet it omits comparative context regarding EVs from other bone niche cells such as osteoblasts, osteocytes, and macrophages. A brief discussion on the roles and distinctions of these EVs in bone remodeling would enrich the analysis.

Figures and plots should be supplemented with explicit labels for each outcome variable analyzed to facilitate reader understanding and to distinguish between structural and functional bone parameters.

**Response**:

Thank you very much for your constructive and insightful suggestions, with which we fully agree. During the initial stages of manuscript design, literature search, and drafting, we focused exclusively on EVs derived from BMSCs and did not include those from other cellular sources such as osteoblasts, osteocytes, osteoclasts, and macrophages. These cells are closely related to the pathophysiology of osteoporosis and the assessment of therapeutic efficacy, and they may directly influence the balance between bone formation and resorption. In response, we have thoroughly supplemented and analyzed the therapeutic effects of EVs derived from BMSCs as well as other cell types in the Discussion section, drawing on relevant literature. This addition aims to enhance the overall quality and readability of the manuscript. We believe that this further elaboration will help to highlight the heterogeneity and distinct roles of various EVs in bone remodeling, thereby deepening readers’ understanding.

Additionally, we have added annotations to Figures 3–10 to indicate the corresponding outcome measures, thereby enhancing the readability and clarity of the manuscript. These improvements aim to help readers more quickly grasp the significance of each figure. The specific revisions and additions can be found in the “Discussion” section, where they have been clearly marked in blue for ease of reference.

EVs derived from various cell types within the osteoimmune microenvironment play a critical role in regulating bone homeostasis, including those from osteoclasts, osteoblasts, and macrophages. Osteoclasts are key contributors to the development and progression of osteoporosis. A previous study demonstrated that EVs derived from osteoclasts promoted the osteogenic differentiation of MSCs *in vitro* by targeting ARHGAP1, a negative regulator of osteogenesis, and exhibited strong bone regenerative capacity in a mouse calvarial defect model (9). Li et al. (10) confirmed that osteoclast-derived exosomal miR-214-3p enhances bone formation in aged OVX mice. The effects of osteoblast-derived EVs are more complex. Davies et al. (11) found that EVs from osteoblasts induced osteogenic mineralization in MSCs, marked by the upregulation of ALP and BMP-2. However, EVs isolated from osteoblasts of osteoporosis patients negatively regulated MSC osteogenic differentiation (12). Moreover, macrophages modulate bone homeostasis through the secretion of cytokines and EVs. EVs from M1 macrophages, enriched in miRNA-155, exacerbate bone loss in osteoporosis models by downregulating DUSP1 and activating the JNK signaling pathway (13). Another study showed that M1 macrophage-derived exosomes promoted the osteogenic differentiation of BMSCs during the early stages of inflammation via microRNA-21a-5p (14). In summary, beyond BMSCs, multiple cell types can influence the osteoporotic microenvironment through EV secretion, yet the diversity and often contradictory effects of these regulatory mechanisms require further mechanistic investigation.

3. The therapeutic potential of EVs in osteoporosis should be discussed in a more balanced manner, including emerging challenges such as EV stability, biodistribution, and dosing strategies in vivo.

**Response**:

Thank you very much for your highly insightful suggestions. While EVs have demonstrated promising potential in the treatment of osteoporosis, several inevitable challenges and unresolved issues remain. These include their stability, biodistribution, accumulation, and metabolism after administration, as well as the impact of different delivery strategies such as intravenous, intraperitoneal, and subcutaneous injection. All of these factors may influence the efficacy and safety of EV-based interventions in osteoporosis models. In response, we have included a dedicated section in the “Discussion” that explores the potential effects of different administration routes, dosages, and frequencies on therapeutic outcomes. We also incorporated findings from previous studies to analyze the in vivo stability, distribution, and metabolism of EVs following injection. Additionally, we further discussed the safety assessment results reported in the included studies. The specific revisions and additions are as follows:

Subgroup analysis based on different EV isolation methods (ultracentrifugation or precipitation kits) indicated that the ultracentrifugation subgroup may enhance BV/TV and Tb.Th while reducing Tb.Sp in the models. Previous studies have shown that conventional isolation techniques such as ultracentrifugation and filtration may cause biomolecular and membrane damage, potentially triggering immune responses (15). Additionally, both ultracentrifugation and precipitation kits can lead to substantial co-precipitation of protein contaminants (16), which may interfere with therapeutic efficacy. These factors may contribute to the significant heterogeneity observed in the analysis of Tb.Sp reduction within the ultracentrifugation subgroup. In contrast, tangential flow filtration achieves reduced EV damage by maintaining controlled shear rates (17). However, none of the ten included studies employed this isolation method. Future research should prioritize standardized protocols for EV isolation and purification to generate higher-quality evidence of therapeutic outcomes.

Additionally, different routes of administration, dosages, and frequencies may influence the biodistribution, accumulation, and metabolism of EVs in vivo. In a mouse model study involving EV intervention, intravenous injection led to primary accumulation in the liver, intraperitoneal injection resulted in significantly increased accumulation in the gastrointestinal tract, and subcutaneous injection showed the lowest liver accumulation. Regarding dosage, both low and moderate doses (<1.0×10^10^ particles/g) predominantly accumulated in the liver, whereas high doses (1.5×10^10^ particles/g) resulted in reduced hepatic accumulation (18). Among the ten included BMSC-EV intervention studies, only one employed intraperitoneal injection. Hematoxylin-eosin staining and biophotonic imaging analyses revealed no apparent systemic toxicity, with EV-loaded nanoparticles mostly cleared by the liver and no reported gastrointestinal accumulation (19). Eight studies used intravenous injection, and two of these reported biosafety data, indicating no observable organ toxicity and no impairment of liver or kidney function (20, 21). Further studies are needed to investigate the metabolic and distributional differences as well as the underlying mechanisms associated with various administration routes, which are crucial for achieving consistent therapeutic efficacy and safety.

**#Reviewer 2**

The manuscript titled: "The Effect of Bone Marrow Mesenchymal Stem Cell-Derived Extracellular Vesicles on Bone Mineral Density and Microstructure in Osteoporosis: A Systematic Review and Meta-Analysis of Preclinical Studies" aims to evaluate the potential role of the BMSCs-derived EVs in the treatment of osteoporosis. The topic of the study is very interesting, even if the author's methodological approach in the assessment of the literature research criteria has driven to several limitations of the study, as themselves very well described before the conclusions. This potential biases are referred to the small number of papers included in the meta-analysis, the presence of a regional risk (all the studies have been conducted in China), few information about the quality of the BMSCs-EVs isolation and characterization, lacking of in vivo models details etc... Nevertheless, the results are precise and very well represented, but some issues must be addressed to reach the journal standard for publication:

1. In the introduction section the authors should open the discussion also to other aspect of the topic, even if not included in their meta-analysis criteria: the BMSCs derived EVs classification in bone tissue studies (in terms of size, distribution, characterization etc...), some specific information about the osteoporosis animal models (Ovariectomy, hormonal intervention, immobilization, dietary manipulation, transgenic models etc...), the presence of extracellular vesicles differently derived in the same tissue niche (from MSCs, osteoblasts, osteoclasts, macrophages...), the diagnostic and therapeutic application of the EVs in osteoporosis disease.

**Response**:

Thank you very much for your positive evaluation of our study and for providing such insightful suggestions for revision. We fully agree with your points and have made careful and thorough modifications accordingly. Based on your recommendations, we have expanded the “Discussion” section to include a more detailed analysis of EV classification, osteoporosis animal models, EVs from different cellular sources, and the diagnostic and therapeutic potential of EVs in osteoporosis.

1) Specifically, regarding the classification of BMSC-derived EVs, our initial manuscript provided only a brief and somewhat vague description, referring to “engineered methods” and “engineered targets.” However, we now recognize that the processes of EV isolation and purification are critical to their functionality, and any variation in these procedures may substantially impact the therapeutic efficacy of EVs in treating osteoporosis. In response, we re-examined the literature and extracted additional data concerning the characteristics of EVs, including their isolation methods, purification techniques, and particle size. In the revised manuscript, we have supplemented the data extraction section with detailed descriptions of these characteristics. The specific additions are as follows:

**Data extraction**

A standardized electronic spreadsheet was used to collect general information from the included authors, year, animal characteristics (species, sample size, modeling methods), EVs characteristics (isolation, purification, and diameter), intervention protocols (EVs administration frequency, routes, and duration), and various outcome indicators (BMD, BV/TV, Tb.N, Tb.Sp, Tb.Th, Ct.Th, and ultimate load-bearing capacity).

The isolation methods for BMSC-EVs included ultracentrifugation (N=7) and precipitation kits (N=2), while one study did not report the purification details. Purification approaches involved filtration through a 0.22/0.45 μm membrane (N=6) and PBS-washed precipitation (N=1); three studies did not specify purification procedures. The particle size range of EVs was 30–500 nm.

Next, we conducted subgroup analyses based on different isolation methods (ultracentrifugation or precipitation kits), purification techniques (filtration through a membrane or pellet washed with PBS), and EV sizes (small EVs or large EVs) to explore potential sources of heterogeneity in key outcome indicators showing significant variability, including BV/TV, Tb.Th, and Tb.Sp. These analyses were performed to identify methodological factors that may contribute to the observed heterogeneity in therapeutic effects. The specific additions and details of this analysis are as follows:

Subgroup analysis indicated that BMSC-EVs significantly improved BV/TV across different animal types (rats or C57 mice), isolation methods (ultracentrifugation), purification approaches (filtration through a 0.22/0.45 μm membrane), EV size (small EVs), intervention frequency (once weekly), and treatment durations (≤4 weeks or >4 weeks) (**Supplementary Figures 1-6**).

Due to significant heterogeneity (*I*² = 78%, *p* < 0.0001), subgroup analysis was conducted to identify significant factors influencing heterogeneity. Subgroup analyses based on six different grouping strategies demonstrated that BMSC-EVs improved Tb.Th in various subgroups, including the rats subgroup, C57 mice subgroup, ultracentrifugation subgroup, filter-based purification subgroup, small EVs subgroup, once-weekly intervention subgroup, ≤4 weeks subgroup, and >4 weeks subgroup (**Supplementary Figures 7-12**). However, none of the subgroups were significant factors influencing heterogeneity.

Due to significant heterogeneity (*I*² = 84%, *p* < 0.00001), subgroup analysis was performed to identify significant factors contributing to the heterogeneity. Subgroup analyses based on six different stratification methods showed that BMSC-EVs consistently reduced Tb.Sp (**Supplementary Figures 13-18**).

In addition, we have supplemented the discussion section with further analysis and commentary based on previous literature regarding the characteristics of different extracellular vesicles (EVs), including isolation methods and vesicle size. We summarized the potential impact of these EV characteristics on the results, aiming to promote standardization of procedures in future research. The specific additions are as follows:

**EV Characteristics and Significant Heterogeneity**

EVs exhibit a broad size range, and the sizes of different types of EVs vary inconsistently. According to the latest MISEV2023 guidelines (22), EVs with diameters less than 200 nm are generally classified as small EVs, although a consensus on the precise threshold has yet to be reached. Based on this classification, we conducted subgroup analyses of BV/TV, Tb.Th, and Tb.Sp for small EVs (<200 nm) and large EVs (≥200 nm). The results suggest that small EVs may play a beneficial role in increasing BV/TV and Tb.Th and in reducing Tb.Sp. Notably, small and large EVs exhibit distinct biodistribution and accumulation patterns in rodent models following administration (23). Specifically, small EVs are initially detected in the liver and kidneys, while large EVs appear first in the lungs. Furthermore, compared with large apoptotic EVs (>1000 nm), small apoptotic EVs (<1000 nm) derived from MSCs more effectively induce macrophage polarization toward the M2 anti-inflammatory phenotype and promote wound healing in diabetic mice (24). These findings provide evidence supporting the role of EV size in modulating osteoimmunological processes related to osteoporosis.

Subgroup analysis based on different EV isolation methods (ultracentrifugation or precipitation kits) indicated that the ultracentrifugation subgroup may enhance BV/TV and Tb.Th while reducing Tb.Sp in the models. Previous studies have shown that conventional isolation techniques such as ultracentrifugation and filtration may cause biomolecular and membrane damage, potentially triggering immune responses (15). Additionally, both ultracentrifugation and precipitation kits can lead to substantial co-precipitation of protein contaminants (16), which may interfere with therapeutic efficacy. These factors may contribute to the significant heterogeneity observed in the analysis of Tb.Sp reduction within the ultracentrifugation subgroup. In contrast, tangential flow filtration achieves reduced EV damage by maintaining controlled shear rates (17). However, none of the ten included studies employed this isolation method. Future research should prioritize standardized protocols for EV isolation and purification to generate higher-quality evidence of therapeutic outcomes.

Additionally, different routes of administration, dosages, and frequencies may influence the biodistribution, accumulation, and metabolism of EVs in vivo. In a mouse model study involving EV intervention, intravenous injection led to primary accumulation in the liver, intraperitoneal injection resulted in significantly increased accumulation in the gastrointestinal tract, and subcutaneous injection showed the lowest liver accumulation. Regarding dosage, both low and moderate doses (<1.0×10^10^ particles/g) predominantly accumulated in the liver, whereas high doses (1.5×10^10^ particles/g) resulted in reduced hepatic accumulation (18). Among the ten included BMSC-EV intervention studies, only one employed intraperitoneal injection. Hematoxylin-eosin staining and biophotonic imaging analyses revealed no apparent systemic toxicity, with EV-loaded nanoparticles mostly cleared by the liver and no reported gastrointestinal accumulation (19). Eight studies used intravenous injection, and two of these reported biosafety data, indicating no observable organ toxicity and no impairment of liver or kidney function (20, 21). Further studies are needed to investigate the metabolic and distributional differences as well as the underlying mechanisms associated with various administration routes, which are crucial for achieving consistent therapeutic efficacy and safety.

2) Relevant content and specific information regarding osteoporosis animal models were not described in the original manuscript. Among the ten included studies, eight employed ovariectomy-induced models, one used mechanical unloading, and one did not specify the modeling method. Therefore, subgroup analysis of key outcome indicators based on different modeling approaches was not feasible. In response, we have summarized various osteoporosis modeling methods in the Discussion section and analyzed the potential impact of these different approaches on the outcomes and effects of BMSC-EVs intervention. The specific additions are as follows:

Currently, common animal models for osteoporosis include OVX-induced models, dexamethasone-induced models, and mechanical unloading models. Among the ten studies included in our analysis, eight employed OVX-induced osteoporosis models. Mechanistically, ovariectomy leads to estrogen deficiency, which in turn results in bone loss, manifesting as reduced bone mass and strength (1). However, due to the inability of rats to achieve full skeletal maturity, OVX models using animals such as rabbits (2), Cynomolgus monkeys (3), and sheep (4) are increasingly being adopted. It is noteworthy that even when using OVX to induce osteoporosis, the timeline of bone loss differs between rats and mice (5, 6), which may lead to variability in the therapeutic efficacy of BMSC-EVs. In dexamethasone-induced osteoporosis models, increased bone resorption and disruption of bone microarchitecture are the primary pathological features. In rat models, this is characterized by discontinuous and thin trabecular bone as well as irregularly eroded endosteal surfaces (7). As for mechanical unloading models, the absence of mechanical stimuli may lead to elevated secretion of inflammatory cytokines and enhanced bone resorption, ultimately resulting in disuse osteoporosis (8). However, the specific mechanisms remain to be fully elucidated. Due to the limited number of studies available, we were unable to draw definitive conclusions or provide detailed interpretations regarding the influence of non-OVX modeling methods on the efficacy of EVs.

3) In addition, we reviewed and collected previous studies involving EVs from alternative sources used to treat osteoporosis models. A systematic summary and analysis of these studies were incorporated into the Discussion section, with the aim of comparing the therapeutic efficacy of BMSC-EVs with that of EVs derived from other sources. Furthermore, regarding the diagnostic and therapeutic applications of EVs in osteoporosis, we provided detailed additions and enhancements in both the Introduction and Discussion sections based on existing literature. The specific supplementary content is as follows:

EVs derived from various cell types within the osteoimmune microenvironment play a critical role in regulating bone homeostasis, including those from osteoclasts, osteoblasts, and macrophages. Osteoclasts are key contributors to the development and progression of osteoporosis. A previous study demonstrated that EVs derived from osteoclasts promoted the osteogenic differentiation of MSCs *in vitro* by targeting ARHGAP1, a negative regulator of osteogenesis, and exhibited strong bone regenerative capacity in a mouse calvarial defect model (9). Li et al. (10) confirmed that osteoclast-derived exosomal miR-214-3p enhances bone formation in aged OVX mice. The effects of osteoblast-derived EVs are more complex. Davies et al. (11) found that EVs from osteoblasts induced osteogenic mineralization in MSCs, marked by the upregulation of ALP and BMP-2. However, EVs isolated from osteoblasts of osteoporosis patients negatively regulated MSC osteogenic differentiation (12). Moreover, macrophages modulate bone homeostasis through the secretion of cytokines and EVs. EVs from M1 macrophages, enriched in miRNA-155, exacerbate bone loss in osteoporosis models by downregulating DUSP1 and activating the JNK signaling pathway (13). Another study showed that M1 macrophage-derived exosomes promoted the osteogenic differentiation of BMSCs during the early stages of inflammation via microRNA-21a-5p (14). In summary, beyond BMSCs, multiple cell types can influence the osteoporotic microenvironment through EV secretion, yet the diversity and often contradictory effects of these regulatory mechanisms require further mechanistic investigation.

2. The authors should add onto the plots or tables in Figures 3 to 10 some labeling in order to understand what parameter the reported results are referred to.

**Response**:

Thank you so much for your valuable suggestions. We have added annotations to Figures 3–10 corresponding to the relevant outcome indicators to enhance the readability and clarity of the manuscript, allowing readers to quickly understand the meaning of each figure. The revised figures have been re-uploaded and replaced accordingly. Please feel free to contact us if any further revisions or additions are needed.

**Journal requirements:**

When submitting your revision, we need you to address these additional requirements.

1. Please ensure that your manuscript meets PLOS ONE's style requirements, including those for file naming. The PLOS ONE style templates can be found at https://journals.plos.org/plosone/s/file?id=wjVg/PLOSOne_formatting_sample_main_body.pdf and https://journals.plos.org/plosone/s/file?id=ba62/PLOSOne_formatting_sample_title_authors_affiliations.pdf

**Response**:

Thank you very much for your suggestions and requirements. In accordance with the journal's guidelines and style, we have carefully revised and refined the relevant content in the resubmitted manuscript. Please feel free to contact us if any further modifications are needed.

2. PLOS requires an ORCID iD for the corresponding author in Editorial Manager on papers submitted after December 6th, 2016. Please ensure that you have an ORCID iD and that it is validated in Editorial Manager. To do this, go to ‘Update my Information’ (in the upper left-hand corner of the main menu), and click on the Fetch/Validate link next to the ORCID field. This will take you to the ORCID site and allow you to create a new iD or authenticate a pre-existing iD in Editorial Manager.

**Response**:

Thank you very much for your reminder. In accordance with your request, we have added the ORCID of the corresponding author, Zhenghong Li, to the system.

3. Please include captions for your Supporting Information files at the end of your manuscript, and update any in-text citations to match accordingly. Please see our Supporting Information guidelines for more information: http://journals.plos.org/plosone/s/supporting-information.

**Response**:

Thank you very much for your suggestions and requirements. In accordance with the journal’s guidelines, we have revised and refined the content, including figure captions and those in the supplementary files. Please feel free to contact us if any further modifications are needed.

4. Your manuscript is a Systematic review or meta-analyses. Please change the article type to 'Research Article', and ensure all submission questions are completed, noting the change in publication fees associated with Research Articles.

**Response**:

Thank you very much for your reminder. As requested, we have updated the article type to “Research Article” in the system.

**We would like to express our heartfelt gratitude once again for your thorough review and valuable comments on this manuscript. Your profound insights have greatly enhanced the rigor and clinical relevance of our study. We have revised the paper according to each of your suggestions, with a particular focus on improving methodological transparency, cautious interpretation of results, and practical guidance for clinical applications. If you have any further suggestions regarding the revisions, we sincerely welcome your feedback. We will fully cooperate to ensure that the manuscript meets the highest standards required by the journal.**

**Very best wishes,**

**Zhenghong Li**

**References**

1.	Komori T. Animal models for osteoporosis. Eur J Pharmacol. 2015;759:287-94.

2.	Wanderman NR, Mallet C, Giambini H, Bao N, Zhao C, An KN, et al. An Ovariectomy-Induced Rabbit Osteoporotic Model: A New Perspective. Asian Spine J. 2018;12(1):12-7.

3.	Jerome CP, Turner CH, Lees CJ. Decreased bone mass and strength in ovariectomized cynomolgus monkeys (Macaca fascicularis). Calcif Tissue Int. 1997;60(3):265-70.

4.	El Khassawna T, Merboth F, Malhan D, Böcker W, Daghma DES, Stoetzel S, et al. Osteocyte Regulation of Receptor Activator of NF-κB Ligand/Osteoprotegerin in a Sheep Model of Osteoporosis. Am J Pathol. 2017;187(8):1686-99.

5.	Li M, Shen Y, Wronski TJ. Time course of femoral neck osteopenia in ovariectomized rats. Bone. 1997;20(1):55-61.

6.	He YX, Zhang G, Pan XH, Liu Z, Zheng LZ, Chan CW, et al. Impaired bone healing pattern in mice with ovariectomy-induced osteoporosis: A drill-hole defect model. Bone. 2011;48(6):1388-400.

7.	El-Mahroky SM, Nageeb MM, Hemead DA, Abd Allah EG. Agomelatine alleviates steroid-induced osteoporosis by targeting SIRT1/RANKL/FOXO1/OPG signalling in rats. Clin Exp Pharmacol Physiol. 2024;51(2):e13832.

8.	Wang W, Zheng X, Wang H, Zuo B, Chen S, Li J. Mechanical Unloading Promotes Osteoclastic Differentiation and Bone Resorption by Modulating the MSC Secretome to Favor Inflammation. Cell Transplant. 2024;33:9636897241236584.

9.	Liang M, Yin X, Zhang S, Ai H, Luo F, Xu J, et al. Osteoclast-derived small extracellular vesicles induce osteogenic differentiation via inhibiting ARHGAP1. Mol Ther Nucleic Acids. 2021;23:1191-203.

10.	Li D, Liu J, Guo B, Liang C, Dang L, Lu C, et al. Osteoclast-derived exosomal miR-214-3p inhibits osteoblastic bone formation. Nat Commun. 2016;7:10872.

11.	Davies OG, Cox SC, Williams RL, Tsaroucha D, Dorrepaal RM, Lewis MP, et al. Annexin-enriched osteoblast-derived vesicles act as an extracellular site of mineral nucleation within developing stem cell cultures. Sci Rep. 2017;7(1):12639.

12.	Niedermair T, Lukas C, Li S, Stöckl S, Craiovan B, Brochhausen C, et al. Influence of Extracellular Vesicles Isolated From Osteoblasts of Patients With Cox-Arthrosis and/or Osteoporosis on Metabolism and Osteogenic Differentiation of BMSCs. Front Bioeng Biotechnol. 2020;8:615520.

13.	Yu L, Hu M, Cui X, Bao D, Luo Z, Li D, et al. M1 macrophage-derived exosomes aggravate bone loss in postmenopausal osteoporosis via a microRNA-98/DUSP1/JNK axis. Cell Biol Int. 2021;45(12):2452-63.

14.	Liu K, Luo X, Lv ZY, Zhang YJ, Meng Z, Li J, et al. Macrophage-Derived Exosomes Promote Bone Mesenchymal Stem Cells Towards Osteoblastic Fate Through microRNA-21a-5p. Front Bioeng Biotechnol. 2021;9:801432.

15.	Walker S, Busatto S, Pham A, Tian M, Suh A, Carson K, et al. Extracellular vesicle-based drug delivery systems for cancer treatment. Theranostics. 2019;9(26):8001-17.

16.	Linares R, Tan S, Gounou C, Arraud N, Brisson AR. High-speed centrifugation induces aggregation of extracellular vesicles. J Extracell Vesicles. 2015;4:29509.

17.	Busatto S, Vilanilam G, Ticer T, Lin WL, Dickson DW, Shapiro S, et al. Tangential Flow Filtration for Highly Efficient Concentration of Extracellular Vesicles from Large Volumes of Fluid. Cells. 2018;7(12).

18.	Wiklander OP, Nordin JZ, O'Loughlin A, Gustafsson Y, Corso G, Mäger I, et al. Extracellular vesicle in vivo biodistribution is determined by cell source, route of administration and targeting. J Extracell Vesicles. 2015;4:26316.

19.	Yang Z, Liu X, Zhao F, Yao M, Lin Z, Yang Z, et al. Bioactive glass nanoparticles inhibit osteoclast differentiation and osteoporotic bone loss by activating lncRNA NRON expression in the extracellular vesicles derived from bone marrow mesenchymal stem cells. Biomaterials. 2022;283:121438.

20.	Gui L, Ye Q, Yu L, Dou G, Zhou Y, Liu Y, et al. Bone-Targeting Peptide and RNF146 Modified Apoptotic Extracellular Vesicles Alleviate Osteoporosis. Int J Nanomedicine. 2024;19:471-88.

21.	Huang B, Su Y, Shen E, Song M, Liu D, Qi H. Extracellular vesicles from GPNMB-modified bone marrow mesenchymal stem cells attenuate bone loss in an ovariectomized rat model. Life Sci. 2021;272:119208.

22.	Welsh JA, Goberdhan DCI, O'Driscoll L, Buzas EI, Blenkiron C, Bussolati B, et al. Minimal information for studies of extracellular vesicles (MISEV2023): From basic to advanced approaches. J Extracell Vesicles. 2024;13(2):e12404.

23.	Kang M, Jordan V, Blenkiron C, Chamley LW. Biodistribution of extracellular vesicles following administration into animals: A systematic review. J Extracell Vesicles. 2021;10(8):e12085.

24.	Zhang X, Yang J, Ma S, Gao X, Wang G, Sun Y, et al. Functional diversity of apoptotic vesicle subpopulations from bone marrow mesenchymal stem cells in tissue regeneration. J Extracell Vesicles. 2024;13(4):e12434.
