# Supplementary material for: The effect of bone marrow mesenchymal stem cell-derived extracellular vesicles on bone mineral density and microstructure in osteoporosis: A systematic review and meta-analysis of preclinical studies
Source: PLoS One. 2025 Jun 30;20(6):e0327011. doi: 10.1371/journal.pone.0327011 (PMC12208419; doi:10.1371/journal.pone.0327011)
Supplement: Peer review history

# Peer review: Decision Letter 0

**DOI:** 10.1371/journal.pone.0327011.r001

**Authors:** Gianpaolo Papaccio

Dear Dr. Li,

Thank you for submitting your manuscript to PLOS ONE. After careful consideration, we feel that it has merit but does not fully meet PLOS ONE’s publication criteria as it currently stands. Therefore, we invite you to submit a revised version of the manuscript that addresses the points raised during the review process.

==============================

 the observed differentiation is a result of true transdifferentiation or stress-induced morphological changes?

The use of morphology and limited neural markers (e.g., Nestin, βIII-tubulin) is not sufficient to confirm neural commitment;

Functional validation (e.g., electrophysiological activity) is absent, which weakens the claim of functional neural differentiation;

The experimental design for the in vivo transplantation lacks proper controls and follow-up time points;

There is insufficient explanation of cell tracking, survival, and integration into host neural tissues;

The number of replicates or biological samples is not always stated;

Please submit your revised manuscript by Jun 26 2025 11:59PM. If you will need more time than this to complete your revisions, please reply to this message or contact the journal office at plosone@plos.org . When you're ready to submit your revision, log on to https://www.editorialmanager.com/pone/ and select the 'Submissions Needing Revision' folder to locate your manuscript file.

- A rebuttal letter that responds to each point raised by the academic editor and reviewer(s). You should upload this letter as a separate file labeled 'Response to Reviewers'.
- A marked-up copy of your manuscript that highlights changes made to the original version. You should upload this as a separate file labeled 'Revised Manuscript with Track Changes'.
- An unmarked version of your revised paper without tracked changes. You should upload this as a separate file labeled 'Manuscript'.

If you would like to make changes to your financial disclosure, please include your updated statement in your cover letter. Guidelines for resubmitting your figure files are available below the reviewer comments at the end of this letter.

If applicable, we recommend that you deposit your laboratory protocols in protocols.io to enhance the reproducibility of your results. Protocols.io assigns your protocol its own identifier (DOI) so that it can be cited independently in the future. For instructions see: https://journals.plos.org/plosone/s/submission-guidelines#loc-laboratory-protocols . Additionally, PLOS ONE offers an option for publishing peer-reviewed Lab Protocol articles, which describe protocols hosted on protocols.io. Read more information on sharing protocols at https://plos.org/protocols?utm_medium=editorial-email&utm_source=authorletters&utm_campaign=protocols .

We look forward to receiving your revised manuscript.

Kind regards,

Gianpaolo Papaccio, M.D., Ph.D.

Academic Editor

PLOS ONE

Journal requirements:

When submitting your revision, we need you to address these additional requirements.

1. Please ensure that your manuscript meets PLOS ONE's style requirements, including those for file naming. The PLOS ONE style templates can be found at

https://journals.plos.org/plosone/s/file?id=wjVg/PLOSOne_formatting_sample_main_body.pdf and

https://journals.plos.org/plosone/s/file?id=ba62/PLOSOne_formatting_sample_title_authors_affiliations.pdf

2. PLOS requires an ORCID iD for the corresponding author in Editorial Manager on papers submitted after December 6th, 2016. Please ensure that you have an ORCID iD and that it is validated in Editorial Manager. To do this, go to ‘Update my Information’ (in the upper left-hand corner of the main menu), and click on the Fetch/Validate link next to the ORCID field. This will take you to the ORCID site and allow you to create a new iD or authenticate a pre-existing iD in Editorial Manager.

3. Please include captions for your Supporting Information files at the end of your manuscript, and update any in-text citations to match accordingly. Please see our Supporting Information guidelines for more information: http://journals.plos.org/plosone/s/supporting-information.

4. Your manuscript is a Systematic review or meta-analyses. Please change the article type to 'Research Article', and ensure all submission questions are completed, noting the change in publication fees associated with Research Articles.

Additional Editor Comments:

Reviewer #1: The manuscript addresses an important topic regarding the therapeutic potential of Wharton's Jelly-derived mesenchymal stromal cells (WJ-MSCs) for neurodegenerative diseases.

The overall rationale and design of the study appear to be well-aligned with the field of regenerative medicine and stem cell therapy. In any case, there are many critical issues that need to be addressed before considering it for publication. In particular:

The study lacks a detailed immunophenotypic profile of the WJ-MSCs used, which is crucial for confirming their identity and purity according to ISCT standards;

No data is provided on MSC multilineage differentiation potential;

The authors claim neural differentiation, but the induction protocol lacks sufficient detail, making it difficult to replicate;

It's unclear whether the observed differentiation is a result of true transdifferentiation or stress-induced morphological changes;

The use of morphology and limited neural markers (e.g., Nestin, βIII-tubulin) is not sufficient to confirm neural commitment;

Functional validation (e.g., electrophysiological activity) is absent, which weakens the claim of functional neural differentiation;

The experimental design for the in vivo transplantation lacks proper controls and follow-up time points;

There is insufficient explanation of cell tracking, survival, and integration into host neural tissues;

The number of replicates or biological samples is not always stated;

The literature review is sparse and does not position the work within the broader stem cell/neural differentiation field.

Reviewer #2: The manuscript titled: "The Effect of Bone Marrow Mesenchymal Stem Cell-Derived Extracellular Vesicles on Bone Mineral Density and Microstructure in Osteoporosis: A Systematic Review and Meta-Analysis of Preclinical Studies" aims to evaluate the potential role of the BMSCs-derived EVs in the treatment of osteoporosis. The topic of the study is very interesting, even if the author's methodological approach in the assessment of the literature research criteria has driven to several limitations of the study, as themselves very well described before the conclusions. This potential biases are referred to the small number of papers included in the meta-analysis, the presence of a regional risk (all the studies have been conducted in China), few information about the quality of the BMSCs-EVs isolation and characterization, lacking of in vivo models details etc... Nevertheless, the results are precise and very well represented, but some issues must be addressed to reach the journal standard for publication:

1) In the introduction section the authors should open the discussion also to other aspect of the topic, even if not included in their meta-analysis criteria: the BMSCs derived EVs classification in bone tissue studies (in terms of size, distribution, characterization etc...), some specific information about the osteoporosis animal models (Ovariectomy, hormonal intervention, immobilization, dietary manipulation, transgenic models etc...), the presence of extracellular vesicles differently derived in the same tissue niche (from MSCs, osteoblasts, osteoclasts, macrophages...), the diagnostic and therapeutic application of the EVs in osteoporosis disease.

2) The authors should add onto the plots or tables in Figures 3 to 10 some labeling in order to understand what parameter the reported results are referred to.

[Note: HTML markup is below. Please do not edit.]

Reviewers' comments:

Reviewer's Responses to Questions

[NOTE: If reviewer comments were submitted as an attachment file, they will be attached to this email and accessible via the submission site. Please log into your account, locate the manuscript record, and check for the action link "View Attachments". If this link does not appear, there are no attachment files.]

While revising your submission, please upload your figure files to the Preflight Analysis and Conversion Engine (PACE) digital diagnostic tool, https://pacev2.apexcovantage.com/ . PACE helps ensure that figures meet PLOS requirements. To use PACE, you must first register as a user. Registration is free. Then, login and navigate to the UPLOAD tab, where you will find detailed instructions on how to use the tool. If you encounter any issues or have any questions when using PACE, please email PLOS at figures@plos.org . Please note that Supporting Information files do not need this step.

# Author comment: Author response to Decision Letter 1

**DOI:** 10.1371/journal.pone.0327011.r002

24 May 2025

Point-by-point response to the editor and reviewers’ comments

#Reviewer 1

In this review Authors provided a valuable and timely systematic evidence on the therapeutic relevance of BMSCs-derived EVs.

The study is interesting and well organized and the findings are clearly presented. In any case, the manuscript requires the following enhancements:

1. The introduction does not sufficiently cover the diversity of preclinical models used to simulate osteoporosis. Including descriptions of OVX, glucocorticoid-induced, and mechanical unloading models would improve the readers’ understanding of the model-dependent outcomes and their translational implications.

Response:

Thank you so much for providing us with the opportunity to revise our manuscript and for your constructive suggestions. As you rightly pointed out, the original "Introduction" section did not comprehensively reflect the diversity of osteoporosis animal models, including OVX, glucocorticoid-induced, mechanical unloading, and genetically engineered models, which may have affected the clarity and coherence for readers. In response, we have added a brief overview of these modeling approaches in the revised "Introduction." Furthermore, we have elaborated in the "Discussion" section on the principles and mechanisms underlying several commonly used osteoporosis models, while emphasizing that the mechanisms of these models remain to be fully elucidated. We also acknowledged the limitations of the studies included and provided perspectives for future research. The specific revisions and additions are as follows:

Current research evidence is primarily derived from animal models, with osteoporosis induction methods including ovariectomy (OVX), hormone induction, mechanical unloading, and genetic engineering approaches (1). Regardless of whether the model is established through surgery, pharmacological agents, dietary manipulation, mechanical means, or genetic modifications, the primary objective remains the induction of bone loss characterized by reduced bone mass and disruption of bone microarchitecture. Therefore, the evaluation of the therapeutic efficacy of BMSC-EVs in osteoporosis models is primarily based on preclinical animal models, and they demonstrate promising therapeutic potential.

Currently, common animal models for osteoporosis include OVX-induced models, dexamethasone-induced models, and mechanical unloading models. Among the ten studies included in our analysis, eight employed OVX-induced osteoporosis models. Mechanistically, ovariectomy leads to estrogen deficiency, which in turn results in bone loss, manifesting as reduced bone mass and strength (1). However, due to the inability of rats to achieve full skeletal maturity, OVX models using animals such as rabbits (2), Cynomolgus monkeys (3), and sheep (4) are increasingly being adopted. It is noteworthy that even when using OVX to induce osteoporosis, the timeline of bone loss differs between rats and mice (5, 6), which may lead to variability in the therapeutic efficacy of BMSC-EVs. In dexamethasone-induced osteoporosis models, increased bone resorption and disruption of bone microarchitecture are the primary pathological features. In rat models, this is characterized by discontinuous and thin trabecular bone as well as irregularly eroded endosteal surfaces (7). As for mechanical unloading models, the absence of mechanical stimuli may lead to elevated secretion of inflammatory cytokines and enhanced bone resorption, ultimately resulting in disuse osteoporosis (8). However, the specific mechanisms remain to be fully elucidated. Due to the limited number of studies available, we were unable to draw definitive conclusions or provide detailed interpretations regarding the influence of non-OVX modeling methods on the efficacy of EVs.

2. The current review focuses exclusively on BMSC-EVs, yet it omits comparative context regarding EVs from other bone niche cells such as osteoblasts, osteocytes, and macrophages. A brief discussion on the roles and distinctions of these EVs in bone remodeling would enrich the analysis.

Figures and plots should be supplemented with explicit labels for each outcome variable analyzed to facilitate reader understanding and to distinguish between structural and functional bone parameters.

Response:

Thank you very much for your constructive and insightful suggestions, with which we fully agree. During the initial stages of manuscript design, literature search, and drafting, we focused exclusively on EVs derived from BMSCs and did not include those from other cellular sources such as osteoblasts, osteocytes, osteoclasts, and macrophages. These cells are closely related to the pathophysiology of osteoporosis and the assessment of therapeutic efficacy, and they may directly influence the balance between bone formation and resorption. In response, we have thoroughly supplemented and analyzed the therapeutic effects of EVs derived from BMSCs as well as other cell types in the Discussion section, drawing on relevant literature. This addition aims to enhance the overall quality and readability of the manuscript. We believe that this further elaboration will help to highlight the heterogeneity and distinct roles of various EVs in bone remodeling, thereby deepening readers’ understanding.

Additionally, we have added annotations to Figures 3–10 to indicate the corresponding outcome measures, thereby enhancing the readability and clarity of the manuscript. These improvements aim to help readers more quickly grasp the significance of each figure. The specific revisions and additions can be found in the “Discussion” section, where they have been clearly marked in blue for ease of reference.

EVs derived from various cell types within the osteoimmune microenvironment play a critical role in regulating bone homeostasis, including those from osteoclasts, osteoblasts, and macrophages. Osteoclasts are key contributors to the development and progression of osteoporosis. A previous study demonstrated that EVs derived from osteoclasts promoted the osteogenic differentiation of MSCs in vitro by targeting ARHGAP1, a negative regulator of osteogenesis, and exhibited strong bone regenerative capacity in a mouse calvarial defect model (9). Li et al. (10) confirmed that osteoclast-derived exosomal miR-214-3p enhances bone formation in aged OVX mice. The effects of osteoblast-derived EVs are more complex. Davies et al. (11) found that EVs from osteoblasts induced osteogenic mineralization in MSCs, marked by the upregulation of ALP and BMP-2. However, EVs isolated from osteoblasts of osteoporosis patients negatively regulated MSC osteogenic differentiation (12). Moreover, macrophages modulate bone homeostasis through the secretion of cytokines and EVs. EVs from M1 macrophages, enriched in miRNA-155, exacerbate bone loss in osteoporosis models by downregulating DUSP1 and activating the JNK signaling pathway (13). Another study showed that M1 macrophage-derived exosomes promoted the osteogenic differentiation of BMSCs during the early stages of inflammation via microRNA-21a-5p (14). In summary, beyond BMSCs, multiple cell types can influence the osteoporotic microenvironment through EV secretion, yet the diversity and often contradictory effects of these regulatory mechanisms require further mechanistic investigation.

3. The therapeutic potential of EVs in osteoporosis should be discussed in a more balanced manner, including emerging challenges such as EV stability, biodistribution, and dosing strategies in vivo.

Response:

Thank you very much for your highly insightful suggestions. While EVs have demonstrated promising potential in the treatment of osteoporosis, several inevitable challenges and unresolved issues remain. These include their stability, biodistribution, accumulation, and metabolism after administration, as well as the impact of different delivery strategies such as intravenous, intraperitoneal, and subcutaneous injection. All of these factors may influence the efficacy and safety of EV-based interventions in osteoporosis models. In response, we have included a dedicated section in the “Discussion” that explores the potential effects of different administration routes, dosages, and frequencies on therapeutic outcomes. We also incorporated findings from previous studies to analyze the in vivo stability, distribution, and metabolism of EVs following injection. Additionally, we further discussed the safety assessment results reported in the included studies. The specific revisions and additions are as follows:

Subgroup analysis based on different EV isolation methods (ultracentrifugation or precipitation kits) indicated that the ultracentrifugation subgroup may enhance BV/TV and Tb.Th while reducing Tb.Sp in the models. Previous studies have shown that conventional isolation techniques such as ultracentrifugation and filtration may cause biomolecular and membrane damage, potentially triggering immune responses (15). Additionally, both ultracentrifugation and precipitation kits can lead to substantial co-precipitation of protein contaminants (16), which may interfere with therapeutic efficacy. These factors may contribute to the significant heterogeneity observed in the analysis of Tb.Sp reduction within the ultracentrifugation subgroup. In contrast, tangential flow filtration achieves reduced EV damage by maintaining controlled shear rates (17). However, none of the ten included studies employed this isolation method. Future research should prioritize standardized protocols for EV isolation and purification to generate higher-quality evidence of therapeutic outcomes.

Additionally, different routes of administration, dosages, and frequencies may influence the biodistribution, accumulation, and metabolism of EVs in vivo. In a mouse model study involving EV intervention, intravenous injection led to primary accumulation in the liver, intraperitoneal injection resulted in significantly increased accumulation in the gastrointestinal tract, and subcutaneous injection showed the lowest liver accumulation. Regarding dosage, both low and moderate doses (<1.0×1010 particles/g) predominantly accumulated in the liver, whereas high doses (1.5×1010 particles/g) resulted in reduced hepatic accumulation (18). Among the ten included BMSC-EV intervention studies, only one employed intraperitoneal injection. Hematoxylin-eosin staining and biophotonic imaging analyses revealed no apparent systemic toxicity, with EV-loaded nanoparticles mostly cleared by the liver and no reported gastrointestinal accumulation (19). Eight studies used intravenous injection, and two of these reported biosafety data, indicating no observable organ toxicity and no impairment of liver or kidney function (20, 21). Further studies are needed to investigate the metabolic and distributional differences as well as the underlying mechanisms associated with various administration routes, which are crucial for achieving consistent therapeutic efficacy and safety.

#Reviewer 2

The manuscript titled: "The Effect of Bone Marrow Mesenchymal Stem Cell-Derived Extracellular Vesicles on Bone Mineral Density and Microstructure in Osteoporosis: A Systematic Review and Meta-Analysis of Preclinical Studies" aims to evaluate the potential role of the BMSCs-derived EVs in the treatment of osteoporosis. The topic of the study is very interesting, even if the author's methodological approach in the assessment of the literature research criteria has driven to several limitations of the study, as themselves very well described before the conclusions. This potential biases are referred to the small number of papers included in the meta-analysis, the presence of a regional risk (all the studies have been conducted in China), few information about the quality of the BMSCs-EVs isolation and characterization, lacking of in vivo models details etc... Nevertheless, the results are precise and very well represented, but some issues must be addressed to reach the journal standard for publication:

1. In the introduction section the authors should open the discussion also to other aspect of the topic, even if not included in their meta-analysis criteria: the BMSCs derived EVs classification in bone tissue studies (in terms of size, distribution, characterization etc...), some specific information about the osteoporosis animal models (Ovariectomy, hormonal intervention, immobilization, dietary manipulation, transgenic models etc...), the presence of extracellular vesicles differently derived in the same tissue niche (from MSCs, osteoblasts, osteoclasts, macrophages...), the diagnostic and therapeutic application of the EVs in osteoporosis disease.

Response:

Thank you very much for your positive evaluation of our study and for providing such insightful suggestions for revision. We fully agree with your points and have made careful and thorough modifications accordingly. Based on your recommendations, we have expanded the “Discussion” section to include a more detailed analysis of EV classification, osteoporosis animal models, EVs from different cellular sources, and the diagnostic and therapeutic potential of EVs in osteoporosis.

1) Specifically, regarding the classification of BMSC-derived EVs, our initial manuscript provided only a brief and somewhat vague description, referring to “engineered methods” and “engineered targets.” However, we now recognize that the processes of EV isolation and purification are critical to their functionality, and any variation in these procedures may substantially impact the therapeutic efficacy of EVs in treating osteoporosis. In response, we re-examined the literature and extracted additional data concerning the characteristics of EVs, including their isolation methods, purification techniques, and particle size. In the revised manuscript, we have supplemented the data extraction section with detailed descriptions of these characteristics. The specific additions are as follows:

Data extraction

A standardized electronic spreadsheet was used to collect general information from the included authors, year, animal characteristics (species, sample size, modeling methods), EVs characteristics (isolation, purification, and diameter), intervention protocols (EVs administration frequency, routes, and duration), and various outcome indicators (BMD, BV/TV, Tb.N, Tb.Sp, Tb.Th, Ct.Th, and ultimate load-bearing capacity).

The isolation methods for BMSC-EVs included ultracentrifugation (N=7) and precipitation kits (N=2), while one study did not report the purification details. Purification approaches involved filtration through a 0.22/0.45 μm membrane (N=6) and PBS-washed precipitation (N=1); three studies did not specify purification procedures. The particle size range of EVs was 30–500 nm.

Next, we conducted subgroup analyses based on different isolation methods (ultracentrifugation or precipitation kits), purification techniques (filtration through a membrane or pellet washed with PBS), and EV sizes (small EVs or large EVs) to explore potential sources of heterogeneity in key outcome indicators showing significant variability, including BV/TV, Tb.Th, and Tb.Sp. These analyses were performed to identify methodological factors that may contribute to the observed heterogeneity in therapeutic effects. The specific additions and details of this analysis are as follows:

Subgroup analysis indicated that BMSC-EVs significantly improved BV/TV across different animal types (rats or C57 mice), isolation methods (ultracentrifugation), purification approaches (filtration through a 0.22/0.45 μm membrane), EV size (small EVs), intervention frequency (once weekly), and treatment durations (≤4 weeks or >4 weeks) (Supplementary Figures 1-6).

Due to significant heterogeneity (I² = 78%, p < 0.0001), subgroup analysis was conducted to identify significant factors influencing heterogeneity. Subgroup analyses based on six different grouping strategies demonstrated that BMSC-EVs improved Tb.Th in various subgroups, including the rats subgroup, C57 mice subgroup, ultracentrifugation subgroup, filter-based purification subgroup, small EVs subgroup, once-weekly intervention subgroup, ≤4 weeks subgroup, and >4 weeks subgroup (Supplementary Figures 7-12). However, none of the subgroups were sig

Attachment Submitted filename: Point-by-point response to the comments.docx

# Peer review: Decision Letter 1

**DOI:** 10.1371/journal.pone.0327011.r003

**Authors:** Gianpaolo Papaccio

The effect of bone marrow mesenchymal stem cell-derived extracellular vesicles on bone mineral density and microstructure in osteoporosis: a systematic review and meta-analysis of preclinical studies

PONE-D-25-21671R1

Dear Dr. Li,

We’re pleased to inform you that your manuscript has been judged scientifically suitable for publication and will be formally accepted for publication once it meets all outstanding technical requirements.

Within one week, you’ll receive an e-mail detailing the required amendments. When these have been addressed, you’ll receive a formal acceptance letter and your manuscript will be scheduled for publication.

An invoice will be generated when your article is formally accepted. Please note, if your institution has a publishing partnership with PLOS and your article meets the relevant criteria, all or part of your publication costs will be covered. Please make sure your user information is up-to-date by logging into Editorial Manager at Editorial Manager®  and clicking the ‘Update My Information' link at the top of the page. If you have any questions relating to publication charges, please contact our Author Billing department directly at authorbilling@plos.org.

If your institution or institutions have a press office, please notify them about your upcoming paper to help maximize its impact. If they’ll be preparing press materials, please inform our press team as soon as possible -- no later than 48 hours after receiving the formal acceptance. Your manuscript will remain under strict press embargo until 2 pm Eastern Time on the date of publication. For more information, please contact onepress@plos.org.

Kind regards,

Gianpaolo Papaccio, M.D., Ph.D.

Academic Editor

PLOS ONE

Additional Editor Comments (optional):

Reviewers' comments:

Reviewer's Responses to Questions

**Comments to the Author**

Reviewer #1: All comments have been addressed

Reviewer #2: All comments have been addressed

**********

2. Is the manuscript technically sound, and do the data support the conclusions?

Reviewer #1: (No Response)

Reviewer #2: (No Response)

**********

3. Has the statistical analysis been performed appropriately and rigorously?

Reviewer #1: (No Response)

Reviewer #2: (No Response)

**********

4. Have the authors made all data underlying the findings in their manuscript fully available?

The PLOS Data policy

Reviewer #1: (No Response)

Reviewer #2: (No Response)

**********

5. Is the manuscript presented in an intelligible fashion and written in standard English?

Reviewer #1: (No Response)

Reviewer #2: (No Response)

**********

Reviewer #1: (No Response)

Reviewer #2: (No Response)

**********

what does this mean? ). If published, this will include your full peer review and any attached files.

If you choose “no”, your identity will remain anonymous but your review may still be made public.

**Do you want your identity to be public for this peer review?** For information about this choice, including consent withdrawal, please see our Privacy Policy

Reviewer #1: No

Reviewer #2: No

**********

# Editor report: Acceptance letter

**DOI:** 10.1371/journal.pone.0327011.r004

**Authors:** Gianpaolo Papaccio

PONE-D-25-21671R1

PLOS ONE

Dear Dr. Li,

I'm pleased to inform you that your manuscript has been deemed suitable for publication in PLOS ONE. Congratulations! Your manuscript is now being handed over to our production team.

At this stage, our production department will prepare your paper for publication. This includes ensuring the following:

* All references, tables, and figures are properly cited

* All relevant supporting information is included in the manuscript submission,

* There are no issues that prevent the paper from being properly typeset

You will receive further instructions from the production team, including instructions on how to review your proof when it is ready. Please keep in mind that we are working through a large volume of accepted articles, so please give us a few days to review your paper and let you know the next and final steps.

Lastly, if your institution or institutions have a press office, please let them know about your upcoming paper now to help maximize its impact. If they'll be preparing press materials, please inform our press team within the next 48 hours. Your manuscript will remain under strict press embargo until 2 pm Eastern Time on the date of publication. For more information, please contact onepress@plos.org.

If we can help with anything else, please email us at customercare@plos.org.

Thank you for submitting your work to PLOS ONE and supporting open access.

Kind regards,

PLOS ONE Editorial Office Staff

on behalf of

Prof. Gianpaolo Papaccio

Academic Editor

PLOS ONE
